# Plant-Based Antidiabetic Nanoformulations: The Emerging Paradigm for Effective Therapy

**DOI:** 10.3390/ijms21062217

**Published:** 2020-03-23

**Authors:** Saikat Dewanjee, Pratik Chakraborty, Biswajit Mukherjee, Vincenzo De Feo

**Affiliations:** 1 Advanced Pharmacognosy Research Laboratory, Department of Pharmaceutical Technology, Jadavpur University, Kolkata 700032, India; pratik.chakraborty88@yahoo.com; 2 Pharmaceutics Research Laboratory, Department of Pharmaceutical Technology, Jadavpur University, Kolkata 700032, India; biswajit.mukherjee@jadavpuruniversity.in; 3 Department of Pharmacy, University of Salerno, 84084 Fisciano, Italy

**Keywords:** bioavailability, diabetes, drug delivery, nanoformulation, natural products, phytochemicals

## Abstract

Diabetes mellitus is a life-threatening metabolic syndrome. Over the past few decades, the incidence of diabetes has climbed exponentially. Several therapeutic approaches have been undertaken, but the occurrence and risk still remain unabated. Several plant-derived small molecules have been proposed to be effective against diabetes and associated vascular complications via acting on several therapeutic targets. In addition, the biocompatibility of these phytochemicals increasingly enhances the interest of exploiting them as therapeutic negotiators. However, poor pharmacokinetic and biopharmaceutical attributes of these phytochemicals largely restrict their clinical usefulness as therapeutic agents. Several pharmaceutical attempts have been undertaken to enhance their compliance and therapeutic efficacy. In this regard, the application of nanotechnology has been proven to be the best approach to improve the compliance and clinical efficacy by overturning the pharmacokinetic and biopharmaceutical obstacles associated with the plant-derived antidiabetic agents. This review gives a comprehensive and up-to-date overview of the nanoformulations of phytochemicals in the management of diabetes and associated complications. The effects of nanosizing on pharmacokinetic, biopharmaceutical and therapeutic profiles of plant-derived small molecules, such as curcumin, resveratrol, naringenin, quercetin, apigenin, baicalin, luteolin, rosmarinic acid, berberine, gymnemic acid, emodin, scutellarin, catechins, thymoquinone, ferulic acid, stevioside, and others have been discussed comprehensively in this review.

## 1. Introduction

Nanoscience and nanotechnology have progressed rapidly in recent years with the scope of various applications. Nanotechnology has spread its applications in several fields including medical and health sciences. In the field of medical science, nanosized formulations offer an unprecedented success in drug delivery systems over conventional formulations via enhancing clinical efficacy of therapeutic agents through improving their biopharmaceutical attributes, pharmacokinetic profiles, and target specificity [1]. The development of nanocarrier-assembled drug nanoparticles, such as polymeric nanoparticles, liposomes, dendrimers, niosomes, nanomicelles, metallic nanoparticles, stimuli-responsive nanoparticles, nanostructured lipid carriers, and nanofabricated devices have been found to produce great success over conventional drug delivery systems in terms of effectiveness, stability, bioavailability, biodistribution, and drug release [1]. Combinations of nanocarriers with ligands make them more targeted with the consequent advantage of the protection of entrapped drugs against degradation. Increased success of nanostructured drug delivery system has gained the interest of many scientists to develop novel formulations against different diseases, such as different types of cancer, inflammatory disorders, cardiovascular diseases, infectious diseases, etc. [2]. Nanoformulations were also found to be efficacious against common metabolic syndrome, such as diabetes mellitus to deliver insulin and oral hypoglycaemic agents. Nanocarrier-assembled nanoparticles of hypoglycaemic agents promote the functionalization of the antidiabetic agents via improving drug penetration to the specific target, prolonging the hypoglycaemic effect, and minimizing the risk of side effects.

Over the years, several pieces of research yielded a number of target-specific small molecules from natural resources including plant phenolics to be effective against diabetes [3]. Many of these have been found to exhibit exciting antidiabetic activity in vitro [3]. However, a gap exists between the in vitro observations and the in vivo effects, which reduce their clinical usefulness [4]. Poor water solubility, rapid metabolism, poor bioavailability, and high P-glycoprotein (P-gp) efflux have been found to be accountable for their in vivo ineffectiveness [3,5]. To overcome the pharmaceutical incompetence of these compounds and to utilize their beneficial effects against diabetes, different pharmaceutical approaches, such as hydrotrophy, micronization, lipid-based emulsion systems, solid dispersions, etc. have been proposed by different scientists over the years [6]. However, nanocarrier-assembled nanosized drug delivery has been found to exhibit remarkable prospects over other dosage forms to deliver naturally occurring antidiabetic agents possessing poor pharmaceutical attributes [1]. Nowadays, the nanodrug delivery system is progressively gaining more attention in the formulation development research to achieve better therapeutic efficacy by reducing dosing frequency, enhancing bioavailability, achieving sustained-release properties, promoting selectivity, and reducing other undesirable biopharmaceutical attributes [6]. This review emphasized the distinctive features of nanoformulations of antidiabetic agents from natural sources.

## 2. Diabetes Mellitus: General Overview

Diabetes mellitus is a serious and pervasive metabolic syndrome characterized by high glucose levels in blood. It can be classified into two major types: type 1 and type 2 diabetes mellitus. Over the past few decades, the prevalence of diabetes has climbed exponentially [7]. In 2014, approximately 422 million people constituting 8.5% of the total global population were diagnosed diabetic [8,9]. Among them, 7% of people with diabetes were categorized as type 2 diabetic patients [9]. It has been predicted that approximately 642 million people will suffer from diabetes mellitus by 2040 [10]. Diabetes is a silent killer which can provoke a number of slowly or rapidly developing pathogenesis, such as nephropathy, retinopathy, neuropathy, cardiomyopathy, peripheral arterial disease, coronary artery disease, and stroke [11,12,13,14,15]. Glycaemic control has been regarded to be the principal therapeutic intervention in diabetes mellitus. However, multiple risk factors in diabetes, lethal complications, and establishment of vasculopathy before diagnosis necessitate developing novel therapeutic strategies for the effective management of diabetes [12]. Insulin, a polypeptide hormone, is secreted from β cells of islets of Langerhans in the pancreas and regulates the uptake and utilization of glucose by the tissues to produce energy [16]. For type 1 diabetic patients, typical treatment includes injections of long-acting insulin to maintain a basal level of insulin, in combination with bolus injections of fast-acting insulin at mealtimes [17]. Several hypoglycaemic agents singly or in combination with insulin are clinically used to treat type 2 diabetes mellitus [9,14]. However, the clinically available antidiabetic agents disappoint both clinicians and patients owing to their untoward effects, which increasingly shifted the focus toward the discovery of novel antidiabetic agents. On the other hand, many naturally occurring phytochemicals have shown huge prospects against diabetes and diabetic complications in preclinical assays via aiming at multiple targets [3,9]. 

## 3. Plant-Derived Small Molecules as Antidiabetic Agents

**Figure 1:**
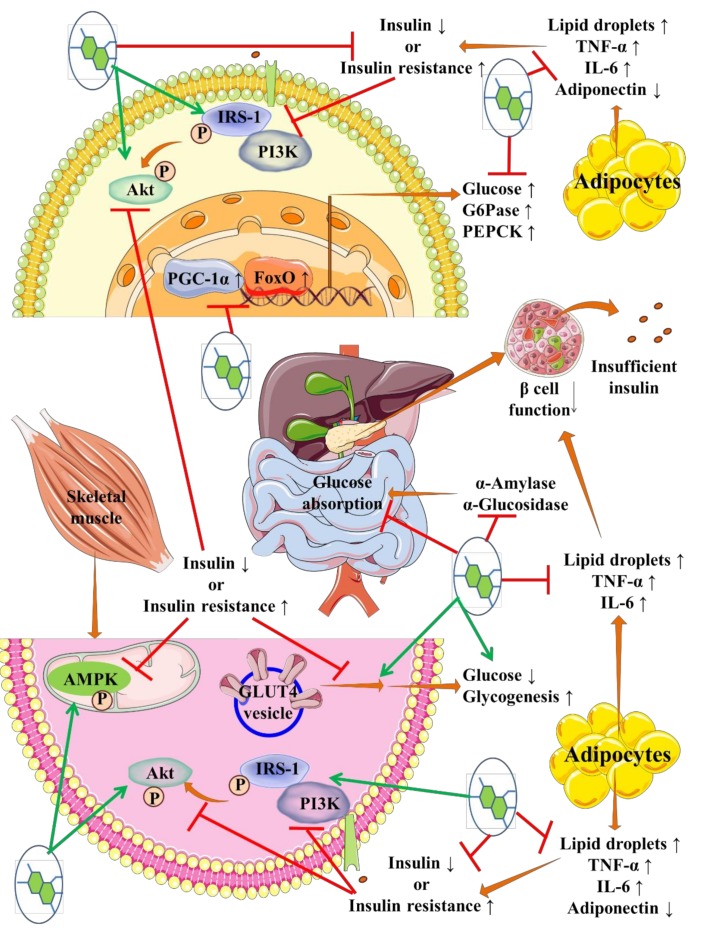
Multiple therapeutic targets of plant secondary metabolites in diabetes management. Orange arrows indicate downstream cellular events; upward arrows indicate upregulation; downward arrows indicate downregulation; green arrows indicate activation; red lines indicate inhibition. Akt: protein kinase A, AMPK: 5′ AMP-activated protein kinase, FoxO: forkhead box protein O, G6Pase: glucose 6-phosphatase, GLUT: glucose transporter, IL: interleukin, IRS-1: insulin receptor substrate-1, PEPCK: phosphoenolpyruvate carboxykinase, PGC-1α: peroxisome proliferator-activated receptor-γ coactivator-1α, PI3K: phosphoinositide 3-kinase, TNF-α: tumour necrosis factor-α.

Many plant-derived secondary metabolites were reported to possess significant antidiabetic activities. Plant secondary metabolites have been revealed to exhibit antidiabetic effect via multiple mechanisms, which include suppression of glucose absorption, restoration of the functional mass of β cells, improvement of insulin expression, reversal of insulin resistance, promotion of glucose utilization, and regulation of carbohydrate and lipid metabolism (Figure 1). 

Several phytochemicals have been found to suppress postprandial hyperglycaemia by interrupting carbohydrate digestion and retarding glucose absorption through inhibition of intestinal carbohydrate digesting enzymes, such as α-amylase, α-glucosidase, and β-glucosidase [18]. Resveratrol, myricitrin, baicalin, apigenin, quercetin, naringenin, curcumin, luteolin scutellarin, ferulic acid, gallic acid, rosmarinic acid, and some anthocyanins can inhibit intestinal carbohydrate-digesting enzymes [18,19,20,21,22,23,24]. 

Progressive functional loss of β cells in diabetic milieu caused by high lipids, high glucose, inflammatory mediators secreted by the adipose tissue, and endoplasmic reticulum stress can decrease insulin secretion, thereby developing persistent hyperglycaemia [25]. Curcumin, gymnemic acids, silymarin, quercetin, resveratrol, and berberine have been proposed to restore functional mass of pancreatic β cells via multiple targeting, thus can attribute to the prospective therapeutic strategy in diabetes [25]. Curcumin and resveratrol can improve β cell function by suppressing pathologic signalling events through inhibition of the phosphodiesterases in β cells [26]. Resveratrol can restore β cell function by endorsing sirtuin 1 (SIRT1) activation, which subsequently endorses pancreatic and duodenal homeobox 1 (PDX1)-triggered insulin expression and abolishes forkhead box O1 (FoxO1)-governed transcriptional actions [27]. Mangiferin can stimulate β cell function via dipeptidyl peptidase-IV (DPP-4) inhibition [28]. Gymnemic acid has been proposed to protect β cells against oxidative damage and stimulate glucose-induced insulin secretion by recruiting glucose transporter type (GLUT)2 signalling [29]. Stevioside can directly target pancreatic β cells and promote insulin secretion [30]. In addition, it improves β cell function by potentiating the transient receptor potential cation channel subfamily melastatin member 5 (TRPM5) channel activity [31]. Asiatic acid can preserve β cell population by endorsing protein kinase B (Akt) and B-cell lymphoma-extralarge (Bcl-xL) expressions [32]. Flavonoids ensure β cell survival against high glucose, high lipids, and pro-inflammatory cytokines by inhibiting nuclear factor kappa-light-chain-enhancer of activated B cells (NF-κB) activation, endorsing phosphoinositide 3-kinase (PI3K)/Akt signalling, inhibiting nitric oxide production, and decreasing oxidative stress [33]. In addition, flavonoids can restore the secretory function of β cells through phospholipase C (PLC), protein kinase C (PKC), protein kinase A (PKA), cyclic adenosine monophosphate (cAMP) regulation [33]. Naringenin can restore the function of β cells via activating GLUT2, PDX1, Akt, insulin receptor substrate (IRS), B-cell lymphoma 2 (Bcl2), and heat shock protein (Hsp)70/90 genes [34]. In addition, it can prevent β cell loss by suppressing pro-apoptotic genes such as Bcl-2-associated X protein (Bax), caspase 3, and acetyl-CoA carboxylase (Acc1) [34]. Silymarin, a flavonoid, was found to endorse β cell neogenesis and insulin production by triggering Nkx6.1 and insulin mRNA activation [35]. Gallic acid prevents high gluco-lipid-induced β cell dysfunction via inhibiting apoptosis and restoring PDX1 and insulin expressions [36].

Insulin resistance primarily hampers glucose utilization in type 2 diabetes [37]. Restoration of insulin signalling is a therapeutic approach to reciprocate insulin resistance [37]. Insulin receptor (IR), insulin receptor substrate (IRS)-1, PI3K, Akt, and GLUT4 are the key components of insulin signalling in skeletal muscle [9,15]. Phosphorylation of IRS1 at Tyr 895 activates PI3K, which subsequently endorses phosphorylation of Akt at Ser 473 and Thr308 resulting in GLUT4 translocation into the membrane [9,15]. GLUT4 translocation can facilitate glucose uptake and utilization. 5′ AMP-activated protein kinase (AMPK) regulates cellular energy homeostasis in this process [9,15]. In addition, phosphorylated Akt can prevent FoxO1 activation via inhibiting the phosphorylation of FoxO1, thus abrogate the expression of hepatic gluconeogenic genes, such as phosphoenolpyruvate carboxykinase (PEPCK), and glucose-6-phosphatase (G6Pase) [38]. AMPK can also suppress PEPCK and G6Pase activities in the liver [39]. Several phytochemicals have been proposed to regulate glucose utilization through stimulation of GLUT4 translocation to membrane, thus improve postprandial glucose uptake [40]. Resveratrol [24,41], curcumin [42], naringenin [34], quercetin [43], apigenin [44], rosmarinic acid [22], berberine [45], stevioside [46], asiatic acid [47], glycyrrhizin [48], gallic acid [49], thymoquinone [50] can elicit insulin-provoked glucose uptake into skeletal muscle by endorsing IRS-1/PI3K/Akt/GLUT4/AMPK signalling. 

Peroxisome proliferator-activated receptor (PPAR) subtypes have been regarded as the key metabolic regulators. PPAR-α and PPAR-β/δ are implicated in fatty acid uptake and fatty acid oxidation; while, PPAR-γ promotes glucose and lipid uptake, glucose oxidation, and insulin responsiveness [51]. Several phytochemicals have been reported to activate PPARs, thereby playing important roles in the management of diabetes. Curcumin [52], berberine [53], glycyrrhizin [54], and scutellarin [55] were found to activate glucose and lipid metabolism by activating PPAR-α in the adipose tissue; while, naringenin [56], myricetin [57], quercetin [58], mangiferin [59], ferulic acid [60], bixin [61], silymarin [62], α-eleostearic acid [63], glycyrrhizin [54], and asiatic acid [64] can endorse hepatic lipid metabolism via endorsing PPAR-α activation. Gymnemic acid was found to endorse fatty acid oxidation and reciprocate insulin resistance in the liver, skeletal muscle and adipose tissue through PPAR-δ activation [65]. Plant phenolics can maintain energy homeostasis by triggering PPAR-δ activation [66]. Several naturally occurring small molecules, such as curcumin [52], resveratrol [67], naringenin [34], luteolin [68], quercetin [43], apigenin [44], emodin [68], berberine [69], baicalin [70], gallic acid [49], and catechin [71] were reported to elicit glucose and lipid metabolism via endorsing transcriptional activity of PPAR-γ in adipose tissue. 

**Figure 2:**
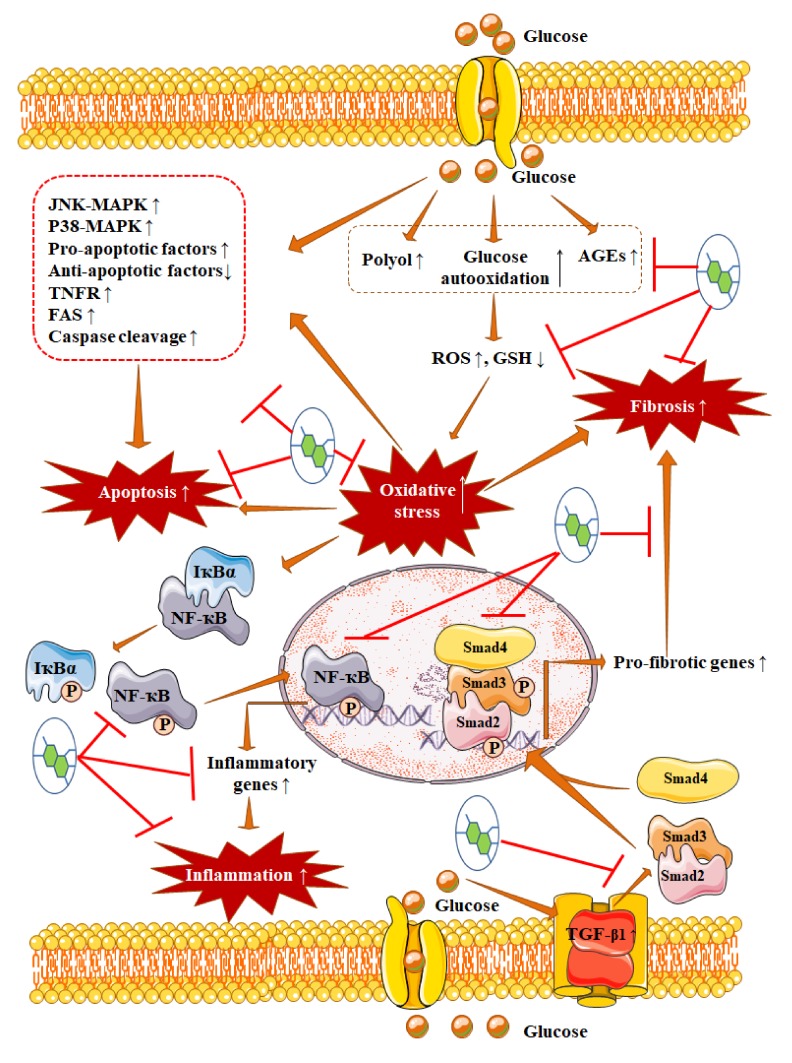
Overall protective mechanism of plant secondary metabolites in diabetic complications. Orange arrows indicate downstream cellular events; upward arrows indicate upregulation; downward arrows indicate downregulation; red lines indicate inhibition. AGEs: advanced glycation end products, FAS: Fas cell surface death receptor, IκBα: nuclear factor of kappa light polypeptide gene enhancer in B-cells inhibitor α, JNK: c-Jun N-terminal kinases, MAPK: mitogen-activated protein kinase, NF-κB: nuclear factor kappa-light-chain-enhancer of activated B cells, ROS: reactive oxygen species, Smad: mothers against decapentaplegic homolog, TGF-β1: transforming growth factor β1, TNFR: tumour necrosis factor receptor.

In addition, plant-derived small molecules have proven their therapeutic effectiveness against diabetes-mediated vascular complications, such as nephropathy, retinopathy, cardiomyopathy, neuropathy, adipose tissue dysfunctions, hepatic disorder, and other vascular diseases via multiple mechanisms. Precisely, hyperglycaemia can elicit oxidative stress by triggering free radical production and impairing endogenous redox defense system [9,12,14,15,72]. Oxidative stress simultaneously endorses inflammation, fibrosis, autophagic dysfunction, and apoptosis via endorsing mitogen-activated protein kinase (MAPK)/PKC/NF-κB/transforming growth factor β1 (TGF-β1)/mothers against decapentaplegic homolog (Smad) 2, 3 and 4/α-smooth muscle actin (α-SMA)/collagen signalling [12,13,14]. In contrast, plant-derived antidiabetic agents can alleviate diabetic complications through glycaemic control, antioxidant mechanism, and inhibition of pathological signal transductions involved in inflammation, fibrosis, and apoptosis (Figure 2). Curcumin [73], resveratrol [74], naringenin [34], quercetin [75], apigenin [76,77], myricitrin [78,79], baicalin [80,81], luteolin [82,83], mangiferin [59,84,85], emodin [86,87], rosmarinic acid [88], berberine [89], stevioside [90,91], asiatic acid [92,93,94], glycyrrhizin [95,96,97,98], baicalin [99,100], silymarin [101], gallic acid [102,103,104], catechins [105,106], thymoquinone [107], and ferulic acid [108,109,110] have been revealed to attenuate diabetic vascular complications via modulating multiple molecular targets. 

Despite the aforementioned plant-derived molecules exhibited excellent opportunity to alleviate diabetes and associated complications in preclinical assays, but poor systemic availability owing to undesirable molecular size, low water solubility, poor lipophilicity, rapid metabolism, less penetrability, and high P-gp efflux largely restricts their clinical usefulness as therapeutic agents in diabetes management [3,111]. Table 1 depicts pharmaceutical incompetence of plant-derived antidiabetic molecules. Thus, formulation designers are actively involved to extract their best therapeutic output through developing novel formulations. In this issue, nanotechnology-based formulation designing has been emerging as a solution to eradicate the pharmaceutical incompetence and improve patient compliance.

## 4. Nanocarrier-Based Drug Delivery: A Contemporary Promise

**Figure 3:**
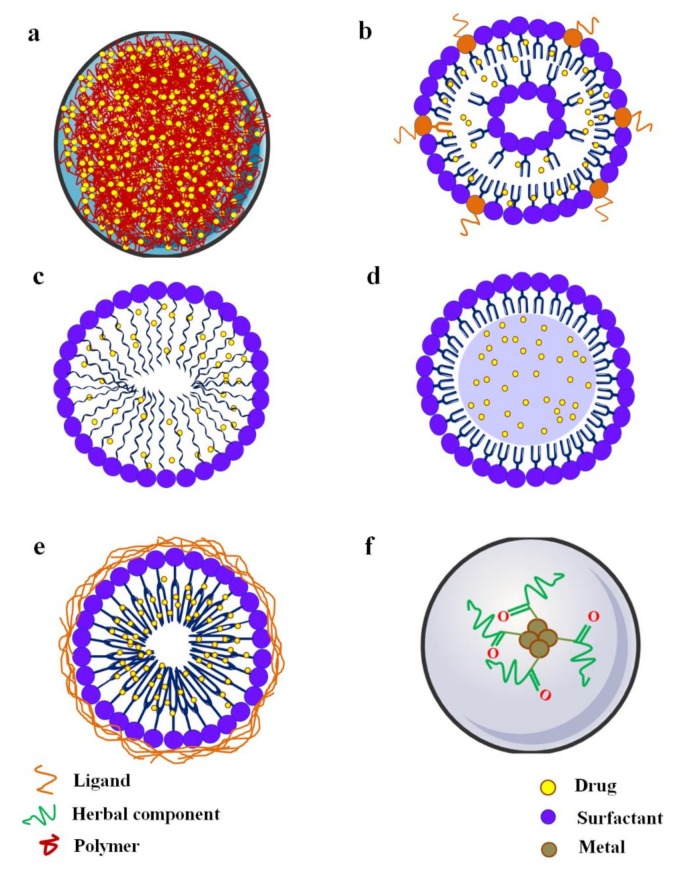
Different types of nanoscale formulations of plant-derived small molecules to achieve better therapeutic efficacy: (**a**) polymeric nanoparticles; (**b**) functionalized capped liposomes; (**c**) non-polymeric micelle; (**d**) solid lipid nanoparticles; (**e**) nanoemulsion; (**f**) herbo-metallic nanoparticles with organic core to improve stability.

**Figure 4:**
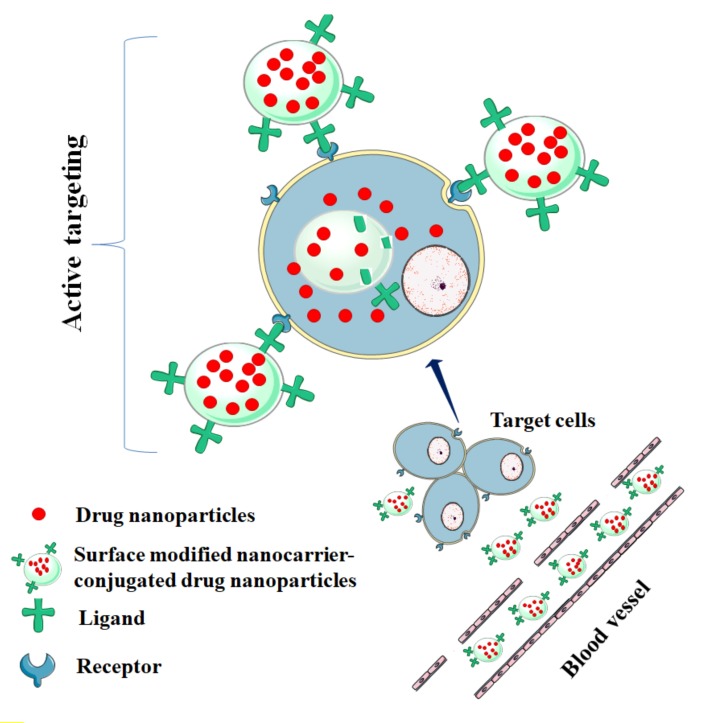
Active targeting by nanocarrier-based formulation by functionalization of their surface with synthetic polymers or conjugating with appropriate ligands. The blue arrow indicates subsequent event.

The prefix ‘nano’ has been derived from Latin ‘nanus’, which means dwarf. Nanoscience deals with the objects with dimensions of 10^−9^–10^−7^ m. In recent years, nanotechnology has gained immense interest in medical science both in diagnosis and in therapy. It has been shown that nanoscaled materials acquire some special physical, chemical and biological properties, which make them attractive for biomedical applications [180]. Curative agents at the nanoscale dimension have been found to break the barrier between therapeutic effects and pharmaceutical incompetence. The development of different types of nanocarriers (Figure 3), such as nanoparticles, liposomes, dendrimers, niosomes, and micelles has become a novel approach in drug delivery over conventional drug delivery systems in terms of effectiveness, stability, bioavailability, target specificity, and release of drugs [181]. Nanocarrier-based drug colloidal nanoparticles with a dimension of less than 500 nm offer a high surface area to volume ratio [181]. Excellent active target specificity of nanocarrier-based formulation can be achieved through functionalization of their surface with synthetic polymers or conjugating with appropriate ligands (Figure 4). Nanocarriers can deliver a wide range of drugs with versatile physicochemical properties [182,183,184]. In contrast, some challenges exist in nanocarrier-based drug delivery, which include poor drug loading capacity, missing cellular uptake ability, toxicity, and questionable biodegradability and ligand-tagging ability [185]. However, formulation scientists have mentioned several approaches to mitigate these limitations and to achieve real-world applications of nanocarrier-based drug delivery. A recent report revealed that the global market of drug nanoformulations is rising progressively with an annual growth rate of 22% [185].

## 5. Nanoformulations in Diabetes Treatment 

Nanotechnology-based approaches offer improved therapeutic management of diabetes mellitus with a minimized risk of acute and chronic complications [1]. A myriad of nanoformulations with varying architectures have been fabricated for the treatment of diabetes mellitus [186]. Nanocarrier-based formulations ensure the efficient delivery of drugs to the target site with the desired release pattern [1,186]. In addition, nanoformulations allow the delivery of drugs through various routes [186,187]. Fabrication of nanocarriers with suitable ligands makes them more targeted and can enhance the systemic availability and stability of drugs. Fabrication of nanocarriers can also reduce the dose of drug and frequency of administration. Finally, fabricated nanoformulations can reduce the risk of toxic manifestations [1,188]. Thus, suitably designed nanoformulations of hypoglycaemic agents may offer improved therapeutic management of diabetes in the near future. The subsequent section of this review emphasized the advancement and effectiveness of nanobased formulations of antidiabetic agents from plant sources.

### 5.1. Curcumin

The development of curcumin nanoformulations has emerged as one of the most prospective approaches to improve solubility, stability, bioavailability, and therapeutic efficacy of curcumin as an antidiabetic agent. Allam and co-workers formulated a curcumin-loaded self-nanophospholipid dispersion using Phosal^®^53 and miglyol 812 at different surfactant ratio [189]. Phosal^®^53 MCT is a phosphatidyl-choline from soybean lecithin, which can serve as an excellent solvent for lipophilic compounds [189]. Curcumin-self-nanophospholipid dispersions were reported to enhance oral bioavailability of curcumin over conventional formulations in rats [189]. Curcumin nanoparticles prepared by a modified emulsion-diffusion-evaporation method was found to reduce fasting blood glucose and glycosylated hemoglobin levels significantly via increasing the expression of insulin and insulin receptor (IR) mRNAs in diabetic rats [190]. Curcumin-ZnO (10 mg/kg, for 21 days) nanoparticles were claimed to be more effective than curcumin nanoparticles (50 mg/kg, for 21 days) in diabetes therapy in terms of reduction of blood glucose, improvement in serum insulin, and activation of GLUT2 and glucokinase genes in pancreas and liver of type 2 diabetic rats [191]. Nanocarrier constituted with poly-(γ-benzyl l-glutamate), poly-(ethylene glycol), and poly-(γ-benzyl 1-glutamate) has been shown to improve the bioactivity and water solubility of curcumin [192]. The design of this nanocarrier offered high loading capacity, gradual release, and low cytotoxicity [192]. Curcumin-encapsulated multi-polymeric nanocarrier achieved better pharmacological effects in cross-regulation of Ca^2+^/calmodulin, calcium-sensing receptor gene and endogenous cystathionine γ-lyase/H_2_S over conventional curcumin formulations in the management of diabetic cardiomyopathy in rats [192]. Curcumin-encapsulated poly(lactic-co-glycolic acid) (PLGA) nanoparticles were found to be effective in enhancing the relative oral bioavailability (5.6-fold to native curcumin) and improving biological half-life of curcumin via improving water solubility, triggering drug release in the intestinal juice, enhancing permeation, inhibiting P-gp efflux, and increasing residence time in the intestinal cavity [193]. Self-nanoemulsification of curcumin has been revealed to prolong plasma acquaintance and improve oral bioavailability of curcumin [194]. Thus, self-nanoemulsified curcumin formulation exhibited better therapeutic effect than native curcumin against experimentally induced diabetic neuropathy in terms of reversing functional, behavioral, and biochemical deficits in rats [194]. Curcumin-entrapped PLGA-PVA (polyvinyl alcohol) nanoparticles were found to improve oral bioavailability of curcumin and thus, achieved better therapeutic effect over free curcumin in delaying cataract formation in diabetic rats [195]. This nanoformulation of curcumin showed superior ability to interfere with the pathological events in the diabetes-mediated cataract formation, such as oxidative stress, polyol pathway, protein insolubilization, protein glycation, and crystallin distribution [195]. Oral delivery of curcumin-loaded pluronic nanomicelles has been shown to attenuate hyperglycaemia, hyperlipidaemia, oxidative stress, and hypoinsulinemia via suppressing β cell damage, promoting β cell regeneration, and triggering the activation of PDX-1 and NK6 homeobox-1 (NKx6.1) genes beyond the values of controls [196]. Devadasu and co-workers formulated curcumin-loaded PLGA nanoparticles for oral delivery, which exhibited improved bioavailability of curcumin and better therapeutic efficacy in the management of hyperlipidaemia, and inflammation in diabetic rats [197]. An amphiphilic polymer prepared by the polymerization of methyaluminoxane (MAO), poly-(ethylene glycol) methacrylate (PEGMA), and 2-(dimethylamino)ethyl methacrylate (DMAEMA) was found to be a potential nanocarrier for encapsulating curcumin [198]. The curcumin-loaded nanoparticles were found to attenuate diabetic neuropathy via suppressing interleukin-1β (IL-1β), connexin43, and phosphorylated protein kinase B (Akt) expressions in dorsal root ganglia and downregulating purinergic 2 (P2) Y12 receptor mRNA expression in satellite glial cells of diabetic rats [198]. Curcumin-loaded polylactic acid (PLA)-polyethylene glycol (PEG) polymeric nanoparticles were found to be effective through the oral route in reciprocating hyperglycaemia, hypoinsulinemia, and diabetes-provoked hepatotoxicity more effectively than free curcumin [199]. Curcumin-PLA-PEG nanoparticles could attenuate hepatotoxicity by mitigating hepatic oxidative stress, inflammation, and fibrosis through suppression of respective signalling events [199]. Curcumin-loaded chitosan nanoparticles were found to improve muscle cell glucose uptake capacity of curcumin in vitro via enhancing its solubility [200]. The curcumin-nanoparticle-loaded topical hydrogel was found to improve aqueous solubility and skin permeability of curcumin [201]. Thus, the nanocurcumin hydrogel improved the wound healing process in diabetic skin of type 1 diabetic rats as compared with normal curcumin hydrogel [201]. In another study, self-assembled curcumin nanoparticles were encapsulated within gelatine microspheres to respond to matrix metalloproteinase 9 (MMP-9) which is commonly over-expressed at diabetic wound sides [202]. Thermosensitive hydrogel in the structure of curcumin-assembled gelatine microspheres was found to improve the capacity of drug release in a sustained manner to the diabetic wound and promoted the efficacy of healing via reducing redox stress and reversing MMP-9-provoked inhibition of cell migration in diabetic mice [202]. Katas and co-researchers formulated a pluronic F-127-based gel containing curcumin-PGT (prostaglandin transporter) DsiRNA chitosan nanoparticles to treat diabetic wounds [203]. Curcumin-loaded chitosan nanoparticles encapsulated into collagen-alginate complex have been designed for the treatment of diabetic wounds. This nanohybrid scaffold offered improvement in stability, porosity, sustainability, biocompatibility, and tissue-regenerating ability to achieve a potential therapeutic option in the management of diabetic wounds [204]. Several clinical trials have reported that nanoformulations of curcumin exhibit improved bioavailability and pharmacokinetic attributes and provided a strong rationale for therapeutic applications of nanocurcumin [205]. In a double-blind randomized clinical trial, type 2 diabetic patients (n = 35) receiving curcumin nanomicelles (80 mg/day) for three months showed a significant reduction in the levels of fasting blood glucose, glycosylated haemoglobin, low-density lipoprotein (LDL)-cholesterol, triglycerides, and body mass index when compared with placebo control patients [206]. In another double-blind randomized placebo-controlled clinical trial, nanocurcumin (80 mg/day, orally) for three months was reported to decrease the levels of fasting blood glucose, glycosylated hemoglobin, triglycerides, LDL-cholesterol, homeostatic model assessment-insulin resistance (HOMA-IR), inflammation markers in the obese patients (n = 42) with nonalcoholic fatty liver disease as compared with placebo control patients [207]. Nonalcoholic fatty liver disease is very closely related to obesity and type 2 diabetes.

### 5.2. Resveratrol

Poor pharmacokinetic and biopharmaceutical properties of resveratrol have been implicated in dose escalation and frequent dosing to achieve desired therapeutic effects [24]. Several strategies, such as chemical modifications, inclusion of bioenhancers, development of resveratrol prodrugs and development of novel pharmaceutical formulations can address the pharmaceutical incompetence of resveratrol [24]. However, nanoencapsulation of resveratrol in lipid nanocarriers, nanoemulsions, micelles, polymeric particles, solid dispersions, and nanocrystals have been demonstrated to be advantageous over other approaches to achieve better stability, improved bioavailability, specific targeting, advanced therapeutic efficacy and improved patient compliance [24,116]. Resveratrol-loaded layer-by-layer nanoformulation consists of 5.5 bilayers of polyallylamine hydrochloride and dextran sulphate and resveratrol nanocores were found to enhance the stability and systemic availability of resveratrol following oral administration [208]. Thus, the formulations represent potential drug delivery tools for resveratrol [208]. Multilayered resveratrol nanoliposome prepared by dry film hydration process and its PEG-amalgamated (PEGylated) modification have been reported to improve glycaemic status, redox status, insulin level in glucose or streptozotocin-treated β-TC cells [209]. The formulations produced a sustained effect up to 24 h in vitro as compared to native resveratrol [209]. Resveratrol-loaded nanocochleates exhibited similar results in glucose or streptozotocin-exposed β-TC cells [210]. Thus, resveratrol-loaded nanoliposome, PEGylated modification of resveratrol-loaded nanoliposome, and resveratrol-loaded nanocochleates could be the effective formulations in the management of type 2 diabetes and associated microvascular complications [209,210]. Resveratrol-loaded casein nanoparticles have been found to be an effective tool for the oral delivery of resveratrol and the formulation exhibited pH resistance, faster penetration, and sustained drug release properties. This formulation was shown to result in a 10-fold increment of oral bioavailability of resveratrol [115]. Resveratrol nanoemulsion (Life Enhancement, California, USA) has been claimed to be effective in reducing hyperglycaemia and oxidative stress in type 2 diabetic rats [211]. Resveratrol-loaded solid lipid nanoparticles have been reported to enhance the therapeutic effect of resveratrol following oral treatment in diabetic rats [212]. The formulation allowed an initial burst followed by a gradual release under normal conditions and improve the oral bioavailability of resveratrol [212]. The formulation has been found to be significantly effective over free resveratrol in reversing insulin resistance through the activation of synaptosomal-associated protein 23 (Snap23), syntaxin-4 (Stx-4), and vesicle-associated membrane protein 2 (Vamp2) mRNAs in muscle and reduction of oxidative stress in sera of type 2 diabetic rats [212]. Resveratrol-loaded PLGA nanoparticles exhibited an excellent and stable delivery with remarkable improvement in encapsulation efficiency, drug loading capacity, solubility, absorption, bioavailability, and sustained release of resveratrol to attenuate nonalcoholic fatty liver disease, which is very closely associated with type 2 diabetes [213]. Galactosylated PLGA has also been found to be a potential nanocarrier for the oral delivery of resveratrol to achieve improved bioavailability and therapeutic efficacy [214]. Oral treatment with resveratrol-assembled gold nanoparticles exhibited excellent inhibitory effects on the activation of vascular endothelial growth factor (VEGF)-1, monocyte chemotactic proteins-1 (MCP-1), intercellular adhesion molecule-1 (ICAM-1), extracellular signal-regulated kinase (ERK) 1/2, NF-κB, TNFα, IL-6, IL-1β genes in the retina of diabetic rats [215]. The nanoformulations could also trigger the activation of retinal pigment epithelium-derived factor (PEDF) mRNA [216]. Thus, resveratrol assembled gold nanoparticles would be a potential therapeutic agent in diabetic retinopathy [215]. To date, there is no such report on the clinical trial of resveratrol nanoformulation on diabetic humans.

### 5.3. Naringenin

Various pharmaceutical strategies, such as formulating with solubility enhancers, P-gp inhibitor, and metabolic inhibitors have been undertaken to improve the pharmacokinetic and biopharmaceutical properties of naringenin in achieving the clinical relevance [118]. However, nanotechnology-based formulation designing has emerged as a prospective strategy over others to eliminate pharmaceutical incompetencies and to achieve better therapeutic efficacy of naringenin. Development of naringenin nanoemulsification using PVP (polyvinylpyrrolidone) K-90 as stabilizer has been found to improve gastrointestinal absorption, dissolution, and oral bioavailability of naringenin over native naringenin [117]. Self-nanoemulsified naringenin delivery system has been found to improve drug release, absorption, and oral bioavailability naringenin as compared to free drug suspension [216]. Naringenin-loaded soluthin-maltodextrin nanocarrier was found to enhance oral bioavailability (116 fold) and to reduce toxicity (21 fold) of naringenin compared to free naringenin [217]. Naringenin-loaded liposomal nanoformulation has been found to increase solubility and oral bioavailability (13.44 fold) of naringenin, thus improving its clinical applications [218]. Despite several nanoformulations of naringenin have been claimed to improve oral bioavailability and therapeutic efficacy against different diseases; however, their potential role in diabetes management is still limited [219]. Naringenin-loaded chitosan core-shell nanoparticles coated with alginate have been found to be effective for oral delivery naringenin ensuring significant drug entrapment (> 90%) and pH-responsive slow and sustained release of naringenin [220]. Naringenin-encapsulated core-shell polymeric nanoparticles were found to exhibit better therapeutic effect than free naringenin in reversing hyperglycaemia, dyslipidaemia, and oxidative stress in diabetic rats [220]. In addition, naringenin-loaded core-shell polymeric nanoparticles have been revealed to be a safe formulation for oral therapy [220].

### 5.4. Quercetin

Nanofabrication of quercetin has offered excellent opportunities in improving oral bioavailability, target specificity, therapeutic efficacy, and compliance. Quercetin-loaded on PLGA nanoparticles was observed to increase the oral bioavailability of quercetin (> five-fold) than free quercetin [221]. This quercetin nanoformulation offered a prolonged release of quercetin up to six days [221]. Thus, the formulation improves therapeutic compliance by reducing the therapeutic dose and frequency of administration [221]. Quercetin-PLGA nanoparticles at the dose of 150 mg/kg in every fifth day could significantly reciprocate hyperglycaemia and oxidative stress (kidney, and pancreas) in diabetic rats and the effect was found to be superior to that of free quercetin at the dose of 150 mg/kg/day [221]. Quercetin nanorods offered an efficient delivery of quercetin with improved pharmaceutical attributes in reciprocating hyperglycaemia, changes in glucose-metabolizing enzymes, and oxidative stress (liver, kidney, and pancreas) in diabetic mice [222]. Nanorods have been proven to enhance the efficiency of diabetes management via improving cellular uptake and bio-distribution of quercetin in the target sites [222]. Quercetin-loaded PEG-block-[poly-(ethylenediaminel-glutamate)-graft-poly-(ε-benzyloxy-carbonyl-l-lysine)] nanocarrier remarkably promoted the therapeutic potential of quercetin than free quercetin in the management of diabetes and associated nephropathy via improving the serum content of quercetin in rats [223]. Quercetin-loaded solid lipid nanoparticles exhibited increase in the absorption and the oral bioavailability of quercetin as compared to native quercetin [223]. Oral delivery of quercetin-succinylated chitosan-alginate core-shell-corona structured nanoparticles remarkably improved oral hypoglycaemic effect of quercetin in diabetic rats compared to native oral quercetin [224]. Quercetin-loaded Soluplus micelles have been found to improve the oral bioavailability (> 16%) of quercetin and maintained a prolonged release pattern in the management of diabetes in rats [225]. Oral delivery of quercetin-conjugated superparamagnetic iron oxide nanoparticles has been found to ameliorate diabetes-provoked memory impairment in rats at a much lower dose compared to free quercetin [226]. Several nanoformulations of quercetin have been claimed to improve oral bioavailability and therapeutic efficacy against diabetes; however, clinical reports on the antidiabetic potential of quercetin nanoformulation are yet to be published.

### 5.5. Apigenin

Several nanoformulations have been proposed to increase the therapeutic efficacy of apigenin, which have not only improved the bioavailability but also simultaneously ensured specific targeting. Microwave-synthesized apigenin-pluronic F127 nanoparticles were found to enhance dissolution rate and oral absorption of apigenin and thus, improved the oral bioavailability to > three-fold compared with the marketed capsule [121]. An apigenin-loaded nanomixed micelles system comprising Soluplus and pluronic F127 polymers has been claimed to improve oral bioavailability (> four-fold), achieve a sustained release, and promote gastrointestinal absorption of apigenin than free apigenin in rats [227]. Apigenin nanomixed micelles significantly improved water solubility and cellular uptake of apigenin [227]. Carbon nanopowder-based solid dispersion of apigenin improved stability and bioavailability of apigenin [228]. Apigenin-loaded nanoliposomes were claimed to inhibit apoptosis of myocardial cells in diabetic cardiomyopathy rats [229]. However, there is no available literature on the antidiabetic efficacy of apigenin nanoparticles till today. 

### 5.6. Myricitrin

Several nanoformulations of myricitrin have been revealed to improve the oral bioavailability and the therapeutic efficacy of myricitrin. The solid lipid nanocarrier system has been found to be an excellent platform for the oral delivery of myricitrin to treat type 2 diabetes and diabetes-provoked oxidative stress in mice [79]. Myricetin-loaded solid lipid nanoparticles achieved a sustained release of myricetin from the formulation and exhibited excellent therapeutic effect in reciprocating hyperglycaemia, insulin resistance, impairment of glucose uptake by myotubes, and apoptosis of pancreas in vitro and in vivo [79]. The myricetin nanoparticles were found to be more effective than that of metformin at much lower dose [79]. The same group has also reported that myricetin-loaded solid lipid nanoparticles can attenuate hyperglycaemia-triggered oxidative stress, inflammation, fibrosis, and apoptosis in high glucose-exposed proximal tubules of mouse in vitro. Thus, the formulation would be equally effective against diabetic nephropathy or other diabetic complications. 

### 5.7. Baicalin

Development of nanoscale formulations of baicalin has emerged as a potential approach to enhance the bioavailability of baicalin in achieving better clinical efficacy [125,230]. Baicalin-entrapped nanoliposome has been reported to be an excellent formulation for the oral delivery exhibiting improved stability, biodistribution and oral bioavailability of baicalin [231]. Similarly, baicalin-loaded nanomicelle containing pluronic P123 copolymer and sodium taurocholate showed improvement of absorption, circulation time and oral bioavailability (> 1.5-fold) of baicalin compared to baicalin suspension in rats and thus can serve as a promising approach of oral delivery of baicalin [232]. Different nanoformulations of baicalin were found to improve oral bioavailability of baicalin; however, only one literature is available regarding the therapeutic benefit of baicalin in diabetes management. Baicalin-loaded nanostructured lipid carriers have been presented as stable for oral delivery offering a sustained release of baicalin and have been proven to increase the antidiabetic efficacy of baicalin [232]. At the same dose, the nanostructured baicalin-lipid complex was found to be more effective to control hyperglycaemia and hyperlipidaemia in diabetic rats as compared with free baicalin and metformin [232]. 

### 5.8. Luteolin

Fabrication of nanoformulation of luteolin has been found to improve the oral bioavailability of luteolin [233,234]. Luteolin-assembled poly(ε-caprolactone)-PLGA-nature oil has been found to be a suitable nanocarrier for increasing its dispersion in the aqueous medium and thereby improve the oral bioavailability [233]. Luteolin-loaded solid lipid nanoparticles were reported to enhance solubility, biological half-life and bioavailability (4.8-fold) of luteolin and thus improved its therapeutic efficacy [235]. However, the effect of luteolin nanoparticles on diabetic animals is yet to be explored.

### 5.9. Mangiferin

The development of nanoparticle-based drug delivery systems has been proposed to be an excellent tool over other mangiferin formulations in improving biopharmaceutical attributes and therapeutic efficacy of mangiferin [5,127]. Mangiferin-loaded self-assembled phospholipidic nanomicelles were reported to improve the biopharmaceutical attributes of mangiferin [127]. Vitamin E-TPGS (D-α-tocopheryl polyethylene glycol 1000 succinate) co-loaded self-assembled phosholipidic nanomixed micelles systems could enhance the intestinal permeability and oral bioavailability of mangiferin [5]. Mangiferin-encapsulated β-lactoglobulin nanoparticles have been formulated to deliver mangiferin through oral route to achieve target specificity, pepsin resistance, protection against the probiotic strains in the intestine, and sustained release properties [236]. However, the effect of mangiferin nanoparticles on diabetes is yet to be explored.

### 5.10. Gymnemic Acid

Nanoscale formulations of gymnemic acid have been found to improve its pharmacokinetic, pharmacodynamic and therapeutic potentials. To enhance the oral bioavailability, Ravichandran developed lyophilized nanocrystals of gymnemic acid, which enhanced the gastrointestinal absorption and oral bioavailability of gymnemic acid [237]. The redispersion of gymnemic acid nanocrystals in water was also claimed to be equally effective and offered improved oral bioavailability of gymnemic acid [238]. The developed gymnemic acid nanocrystals-loaded tablets were found to exhibit significant antihyperglycaemic activity and even produced considerable hypoglycaemia in diabetic and glucose-loaded rats [129]. Gymnemic acid nanocrystals-loaded tablets were able to reduce blood glucose levels in diabetic rats after 3 h of treatment [129]. Biocompatible gymnemic acid-reduced gold nanoparticles have been found to enhance the glucose uptake capacity of 3T3-L1 adipocytes through the insulin-dependent/independent pathway than free drug and the glucose uptake efficacy was found to be similar to that of pioglitazone [239]. In addition, gymnemic acid-reduced gold nanoparticles exhibited significant stability and low toxicity at the therapeutic doses in vitro [239]. Thus, gymnemic-acid-reduced gold nanoparticles would be a potential oral formulation for the treatment of diabetes. Gymnemic-acid-chitosan nanoparticles prepared by emulsion-droplet coalescence method allowed a controlled release of gymnemic acid continuously for 24 h and claimed to be effective in the management of diabetes mellitus [240]. However, more research is required to formulate a potential antidiabetic nanoformulation of gymnemic acid to achieve better antidiabetic efficacy. 

### 5.11. Emodin

Several pharmaceutical attempts were taken to improve the biopharmaceutical potential of emodine; however, emodine nanoformulations have emerged as the most promising approach to improve compliance and bioavailability of emodin. Emodin-loaded nanoemulsion has been found to improve oral bioavailability of emodin effectively via prolonging its residence time, improving tissue distribution, and reducing clearance [241]. Several emodin nanoformulations enhanced the biopharmaceutical competence of emodin for oral delivery. However, adequate literation is not available to reveal the potential application of emodin nanoformulation in diabetes management. Emodin-loaded poly-PEGMA-DMAEMA-MAM nanoparticles have been found to attenuate diabetes-provoked neuropathic pain via suppressing purin 2X3 (P2X3) receptor expression, TNF-α level, and ERK1/2 activation in dorsal root ganglia of type 2 diabetic rats [242].

### 5.12. Rosmarinic Acid

Several nanoformulations can successfully address the major pharmaceutical obstacles of rosmarinic acid. Madureira and co-workers formulated physico-chemically stable and biocompatible solid lipid nanoparticles of rosmarinic acid using carnauba waxes [132]. The same group also formulated rosmarinic acid-assembled solid lipid nanoparticles using carnauba wax [132]. Both the nanoformulations were found to be safe and biocompatible for the oral delivery of rosmarinic acid. In addition, the rosmarinic acid-solid lipid nanoparticles did not exhibit any sign of genotoxicity and cytotoxicity in vitro [132]. Polyacrylamide-cardiolipin-PLGA grafted with surface 83-14 monoclonal antibody has been designed as a potential nanocarriers to deliver rosmarinic acid to the brain for protecting β-amyloid-insulted neurons [243]. Similarly, polyacrylamide-chitosan-PLGA grafted with cross-reacting material 197 and apolipoprotein E nanocarriers was found to be effective in delivering rosmarinic acid to the brain for alleviating neurotoxicity-mediated through β-amyloid [244]. Both these nanocarriers allowed rosmarinic acid to cross blood-brain barrier, improve cellular uptake, and attenuate β-amyloidosis. An earlier report revealed that diabetes neuropathy is often associated with an increase in β-amyloid in neurones [245]. Thus, the therapeutic effect of both these formulations may be evaluated against diabetic neuropathy. Rosmarinic acid-chitosan nanoparticles and rosmarinic acid-loaded solid lipid (Witepsol H15) nanoparticles have been found to exhibit excellent thermal stability, in vitro release, and efficient antioxidant effect without incidence of aggregation [246,247]. Instillation of rosmarinic acid-chitosan-sodium tripolyphosphate nanoparticles have been found to enhance the retention of formulation on ocular mucosa and claimed to be an effective drug delivery system to attenuate oxidative damage in the eyes without exhibiting any toxicity on retinal and corneal cells [248]. Rosmarinic acid-assembled chitosan nanoparticles-loaded hydrogel is a biocompatible topical dosage form, which exhibited a higher degree of wound healing than the rosmarinic acid -gel formulation [248]. In addition, rosmarinic acid-chitosan nanoparticles-loaded hydrogel allows sustained release of drug release for a period of 14 h [248]. Considering antioxidant, compatible and sustained release properties, rosmarinic acid-chitosan nanoparticles may be evaluated against diabetic retinopathy and wound. To date, no report is available to expose the effectiveness of rosmarinic acid nanoformulation on diabetes. However, considering the improvement of therapeutic effectiveness, biopharmaceutical attributes, and pharmacokinetic profiles of different rosmarinic acid nanoformulations for treating various pathological events, they may be subjected to preclinical assays against diabetes and diabetic complications.

### 5.13. Berberine

Several pharmaceutical tactics, such as formulation with permeability enhancers, P-gp inhibitors, muco-adhesive agents have been attempted to eradicate pharmaceutical incompetence of berberine; however, application of nanotechnology has been found to be the most promising tool to improve oral bioavailability and therapeutic efficacy of berberine [249]. Dendrimers-encapsulated berberine, berberine-assembled polymer-lipid hybrid nanoparticles, and chitosan-coated nanoliposomes of berberine have been reported to facilitate oral delivery of berberine with improved pharmaceutical attributes [250,251,252]. Berberine-loaded anhydrous reverse micelle formulated by lyophilisation of water in oil (W/O) emulsion employing soy phosphatidylcholine as emulsifier has been found to improve the oral bioavailability and hypoglycaemic efficacy of berberine [253]. Berberine-assembled solid lipid nanoparticles have been found to enhance oral bioavailability, stability and antidiabetic potential of berberine compared to the free drug [253]. Oral administration of nanoparticles remarkably reduced hyperglycaemia, body-weight gain, and insulin resistance in type 2 diabetic mice compared to native berberine [254]. In addition, berberine-assembled solid lipid nanoparticles have been found to achieve maximum drug concentration in the liver (~ 20-fold higher than in plasma) and significantly attenuated diabetes-provoked hepatosteatosis in type 2 diabetic mice [255]. Ortho-hexadecyl-dextran-encapsulated berberine chloride nanoparticles exhibited an improved bioavailability and controlled release of berberine [256]. The nanoberberine was found to alleviate hyperglycaemic stress in murine hepatocytes via inhibiting glucose-provoked ROS production, oxidative stress, and apoptosis [256]. The nanoberberine formulation was found to exhibit a similar effect as free berberine but at much (~ 20-fold lower) lower concentration [256]. The study implicated the therapeutic usefulness of berberine nanoformulation against diabetic complications at a much lower dose. Berberine-TPGS nanosuspension has been found to achieve better hypoglycaemic potential than native berberine in diabetic mice [257]. Berberine-assembled nanostructured lipid carriers coated with selenium have been found to trigger therapeutic efficacy of berberine in diabetes management than berberine-assembled nanostructured lipid carriers and berberine solution [258]. Selenium modification of nanostructured lipid formulation enhanced intestinal absorption, oral bioavailability, and controlled release of berberine [258]. In addition, selenium-modified nanostructures enhanced the transport of berberine into the enterocytes to achieve better glucose uptake in diabetic rats [258]. Oral delivery of berberine nanoformulations has already been proven to be preclinically effective against diabetes and subsequent complications; however, the extensive clinical investigation is required to deliver effective nanoformulations of berberine for therapeutic purpose.

### 5.14. Stevia Glycosides

*Stevia* glycosides possess impressive antidiabetic effect in preclinical assays; however, they could not impress in clinical studies [140]. It has been revealed that the doses were not sufficient to achieve anticipated pharmacological effect [140], which may be correlated to that of poor bioavailability issue of *Stevia* glycosides. Over the year, several nanoformulations of *Stevia* glycosides have been formulated, which were found to enhance the oral bioavailability, target specificity, and therapeutic efficacy of the *Stevia* glycosides [111]. Stevioside-assembled PEG-PLA nanoparticles achieved a burst release of stevioside at 2 h followed by a controlled release up to 21 days and have been claimed to be safe and effective for the management of diabetes [111]. Pluronic-F-68 copolymer-based stevioside- PLA nanoparticles have been claimed to be an effective antidiabetic nanoformulation for oral delivery [138]. The formulation was found to increase stability, intestinal absorption, biocompatibility, and oral bioavailability of stevioside with an additional success of achieving sustained release pattern [138]. Rebaudioside A-PLA nanoparticles have been claimed to be superior antidiabetic nanoformulation than stevioside-PLA nanoparticles due to their capacity of high drug-loading and sustained release of rebaudioside A [259]. In addition, rebaudioside A has been reported to possess nanocarrier-alike characteristics by self-assembling into micelles in aqueous solutions. Thus, encapsulation of hydrophobic hypoglycaemic agents in rebaudioside A nanomicelles would improve the bioavailability of hydrophobic drugs and can intensify the antidiabetic potential through synergy [260]. 

### 5.15. Asiatic Acid

Application of nanotechnology has been found to be an effective tool to alleviate pharmaceutical limitations, improve compliance, and achieve better therapeutic efficacy of asiatic acid [92]. Asiatic acid tromethamine salt-loaded solid lipid nanoparticles have been found to improve the oral bioavailability (2.5-fold) of asiatic acid than free asiatic acid in rats [141]. PEGylated asiatic acid-loaded nanostructured lipid carriers exhibited enhanced penetration and transport capacities of asiatic acid in the small intestine of rats [261]. The PEG-modified nanoformulation was found to improve oral bioavailability of asiatic acid evidenced from the increase (~ two-fold) in elimination half-life [261]. However, the effect of asiatic acid nanoparticles on diabetes is yet to be explored.

### 5.16. Glycyrrhizin

Various non-vascular administration routes have been proposed to improve the bioavailability of glycyrrhizin; however, the development of nanoscale formulation most suitably addressed the biopharmaceutical incompetence of glycyrrhizin. Incorporation of glycyrrhizin in insulin-loaded poly(ethylcyanoacrylate) nanospheres has been reported to enhance oral absorption of insulin by inhibiting proteolytic enzymes in the digestive tract [262]. The presence of glycyrrhizin may simultaneously synergize the antidiabetic effect of insulin. Glycyrrhizin-assembled sodium deoxycholate-phospholipid-mixed nanomicelles have been proven to be a good nanoformulation for oral delivery of glycyrrhizin with improved pharmacokinetic attributes [142]. Glycyrrhizin-assembled chitosan-gum-arabic nanoparticles have been reported to achieve improved bioavailability and sustained release property [263]. The polymeric nanoformulation could entrap ~ 25% of pure glycyrrhizin [263]. Glycyrrhizin nanoparticles were found to be more effective at a much lower dose in the management of hyperglycaemia and dyslipidaemia in type 2 diabetic rats [263]. However, glycyrrhizin nanoparticles in combination with thymoquinone nanoparticles have been revealed to exhibit better therapeutic efficacy in the management of hyperglycaemia and dyslipidaemia in type 2 diabetic rats than glycyrrhizin nanoparticles [264].

### 5.17. α-Eleostearic Acid

Development of a redox stabilized nanoformulation of α-eleostearic acid or bitter gourd oil can improve its stability and therapeutic efficacy of α-eleostearic acid [144]. α-Eleostearic acid-enriched nanoemulsion of bitter melon seed oil has been found to enhance absorption, biocompatibility, cellular uptake, bioavailability, and antioxidant effect more efficiently than conventional emulsion of α-eleostearic acid [265,266]. In addition, the nanoemulasion exhibited excellent improvement in stability up to 12 weeks as evidenced from the restoration of particle size distribution, hydrodynamic mean diameter, and zeta potential [265,266]. Nanoemulsion of α-eleostearic acid-enriched bitter melon seed oil has been found to reduce blood glucose level and hyperglycaemia-triggered oxidative stress more effectively than the conventional emulsion in type 1 diabetic rats [265]. In another report, nanoemulsion of α-eleostearic acid-enriched bitter melon seed oil showed better cellular penetration to inhibit oxidative stress and inflammation in ex vivo and in vivo models [144]. Thus, nanoemulsion of α-eleostearic acid-enriched bitter melon seed oil would be an improved therapeutic formulation to tackle diabetes and associated toxicosis.

### 5.18. Scutellarin

Scutellarin-loaded hydroxypropyl-β-cyclodextrin-chitosan nanoparticles have been found to enhance drug loading efficiency, solubility, bioavailability, and tissue uptake of scutellarin and thus, be proven to be a potential drug delivery system to achieve a therapeutic effect at the target site [146]. Scutellarin-loaded bovine serum albumin nanoparticles have been found to enhance bioavailability and to achieve sustained release profile of scutellarin following parenteral administration [267]. Scutellarin-loaded amphiphilic chitosan derivatives nanoparticles were found to enhance oral bioavailability (roughly two- to three-fold), elimination half-life, and cellular uptake of scutellarin than free scutellarin [268]. In addition, the nanoformulation could attenuate diabetic retinopathy via suppressing VEGF, VEGF receptor 2 (VEGFR2), and von Willebrand factor (vWF) expressions without affecting hyperglycaemia [268]. However, more research is required to optimize a suitable nanoformulation for the oral delivery of scutellarin.

### 5.19. Silybum Flavonolignans

Considering the poor bioavailability of *Silybum* flavonolignans, they require an escalation in the dose and dosing frequency to achieve desirable therapeutic effects [148]. On the other hand, higher doses of silymarin have been contraindicated in diabetes treatment as high doses of silymarin have been proposed to induce insulin resistance [148]. Further, *Silybum* flavonolignans can impart antidiabetic effects through the liver- and pancreas-centric mechanisms [148,269]. Thus, to attain desire therapeutic efficacy, special attention is required not only to improve the biopharmaceutical and pharmacokinetic aspects but also to ensure target specificity. In these regards, nanoformulations can serve better to reverse the biopharmaceutical and pharmacokinetic limitations of *Silybum* flavonolignans to achieve better compliance and therapeutic efficacy at much lower dose [270]. Silymarin-loaded Soluplus-TPGS nanomicelles have been found to be suitable for oral delivery as the silymarin nanomicelles exhibited improved water solubility, biological stability, P-gp inhibition, gastrointestinal absorption, and cellular uptake of silymarin [271]. Silymarin-PLGA nanoparticles synchronized as microencapsulation, silymarin-loaded solid lipid nanoparticles, and silymarin-loaded nanostructured lipid carriers have also exhibited better pharmacokinetic attributes than silymarin [272,273]. Silymarin-loaded PLGA-pluronic nanomicelles have been reported to be an efficacious oral formulation to treat diabetes and associated complications than free silymarin [148]. The therapeutic effect of silymarin nanomicelles was found to be associated with the activation of insulin, PDX1 and Nkx6.1 genes, inhibition of β cell apoptosis, stimulation of β cell regeneration, suppression of oxidative stress, and reciprocation of dyslipidaemia [148]. The therapeutic superiority of silymarin nanomicelles has been correlated to that of improved in vivo bioavailability, and the sustained release profile of silymarin [148]. Chitosan-modified silybin-loaded solid lipid nanoparticles were found to possess good stability, pronounced mucoadhesive property, sustained release, better absorption, and enhanced cellular uptake of silybin following oral absorption [274]. Carbon nanotube-based drug delivery system and lipid-silybin conjugate nanoparticles have also been found to offer better pharmacokinetic attributes of silybin [275,276]. Pluronic F-68-PLGA-based chitosan-assembled silybin nanoparticles have achieved an improved therapeutic outcome in the management of diabetes than native silybin via enhancing bioavailability, improving tissue targeting, and attaining a sustained release profile of silybin [269]. This engineered nanoformulation significantly reciprocated hyperglycaemia, dyslipidaemia, formation of advanced glycation end products (AGEs), and oxidative stress with superior effect as compared with free silybin [269]. However, extensive research is required to formulate clinically effective nanoparticles of *Silybum* flavonolignans for the management of diabetes mellitus. 

### 5.20. Gallic Acid

Various nanostructured systems have been proven to be prospective delivery strategies to improve the bioavailability and therapeutic efficacy of gallic acid [277,278]. Gallic-acid-encapsulated chitosan nanoparticles showed improvement in α-glucosidase inhibitory effect and would be a suitable antidiabetic nanoformulation [279]. Hydroxyapatite nanoparticles carrying insulin and gallic acid have been found to be an effective oral formulation to mitigate hyperglycaemia [280]. The insulin–gallic-acid nanoparticles were found to improve hepatic glucose utilization via triggering PI3K/Akt/GLUT4 activation in vitro [280]. The developed formulation has been claimed to be a stable, non-toxic, and efficacious agent for oral delivery of insulin-gallic acid combination for the management of diabetes [280].

### 5.21. Catechins

Earlier reports revealed the nanoscale formulations of catechins can improve stability, gastrointestinal absorption, bioaccumulation, oral bioavailability, and therapeutic efficacy of catechins [150,281,282,283,284,285]. Catechin-grafted inulin and catechin-grafted chitosan nanoparticles were reported to exhibit improved antidiabetic potential in terms of α-glucosidase and α-amylase inhibitory effects than free catechins and acarbose [286,287]. In addition, catechin-grafted chitosan nanoparticles exhibited better antioxidant potential than the native catechins [287]. Solid lipid nanoparticles and nanostructured lipid carriers have been developed to enhance solubility, stability, sustained release capacity, and cyto-compatibility of epigallocatechin-3-gallate [288]. Epigallocatechin-3-gallate-loaded chitosan-peptides nanoparticles improve cellular uptake and antioxidant potential than the native of epigallocatechin-3-gallate than free drug [289]. Epigallocatechin gallate-loaded cationic lipid nanoparticles have been reported to exhibit promising effects to mitigate ocular inflammation and oxidative stress, which revealed the probable effectiveness of epigallocatechin-3-gallate in diabetic complications [290]. In addition, self-assembled gelatin-epigallocatechin gallate nanoparticles were found to inhibit ocular angiogenesis remarkably via targeting integrin αvβ3 [291]. The aforementioned reports predicted that the efficacious roles of catechins nanoformulations against diabetes and associated complications. However, extensive research is required to develop a novel catechin nanoformulation to achieve better therapeutic management in diabetes.

### 5.22. Pelargonidin

Development of pelargonidin nanostructured formulations has been anticipated to address the biopharmaceutical and pharmacokinetic incompetence of pelargonidin. Roy and co-workers formulated pelargonidin-assembled PLGA nanoparticles to improve the stability and therapeutic efficacy of pelargonidin [292]. Intravenous injection of pelargonidin-PLGA nanoparticles at an interval of three days was found to be effective in alleviating hyperglycaemia, dyslipidaemia, and oxidative stress in diabetic rats [292]. Pelargonidin-PLGA nanoparticles exhibited a better therapeutic effect compared to native pelargonidin [292]. Pelargonidin-PLGA nanoparticles have also been found to attenuate hyperglycaemia-triggered apoptosis, oxidative stress, DNA damage, and impairment in glucose utilization in L6 skeletal muscle cells more efficiently and at a much low dose (~ 10-fold less) than native pelargonidin [293,294]. In addition, pelargonidin-PLGA nanoparticles exhibited more stability and sustained release profile of pelargonidin [293,294]. Thus, pelargonidin-PLGA would have the possibility in advanced management of diabetes and associated complications.

### 5.23. Thymoquinone

Several thymoquinone nanoformulations have been recommended, which can eliminate its poor pharmaceutical qualities to achieve better therapeutic efficacy. Thymoquinone-loaded pluronic F127 nanoparticles, PLGA-PEG-thymoquinone nanoparticles and thymoquinone-assembled mesoporous silica core-shell nanoformulation were found to exhibit better biopharmaceutical attributes and suitable for sustainable delivery of thymoquinone [295,296,297]. Thymoquinone-assembled PEGylated chitosan nanocapsules have been found to be a non-toxic formulation toward normal cells with improved stability and bioavailability of thymoquinone [298]. Thymoquinone-loaded gum rosin nanocapsules have been found to reverse hyperglycaemia, dyslipidaemia, and glycosylation of haemoglobin more efficiently at a much lower dose than metformin, metformin nanoformulation, and free thymoquinone [153]. In addition, thymoquinone nanoformulation achieved a sustained release profile of thymoquinone to improve the therapeutic efficacy [153]. However, the combination of glycyrrhizin and thymoquinone nanoparticles has been found to exhibit better therapeutic efficacy in the management of hyperglycaemia and dyslipidaemia in type 2 diabetic rats than thymoquinone nanoparticles [264]. Thymoquinone-loaded nanosized liposomes more efficiently alleviated hyperglycaemia, hepatoxicity, and nephrotoxicity in the diabetic mice than native thromboquinone [299]. The formulation restored the structural integrity of pancreatic β cells [300]. In addition, thymoquinone liposomes were found to alleviate systemic candidiasis in diabetic mice [299]. Thymoquinone-encapsulated PLGA nanoparticles exhibited a sustained release of thymoquinone over a period of seven days and preserved the antioxidant profile of thymoquinone [300]. Thus, thymoquinone-PLGA nanoparticles would be efficient to deliver thymoquinone to mitigate diabetic complications.

### 5.24. Ferulic Acid

The pharmaceutical strategies in developing ferulic acid nanoformulation have been found to mitigate physicochemical, biopharmaceutical, and pharmacokinetic incompetence of ferulic acid. Ferulic-acid-assembled chitosan-coated PLGA nanoparticles have been found to improve intestinal permeability, gastrointestinal stability, biological half-life, and oral bioavailability of ferulic acid [301]. The mucoadhesive property of ferulic-acid-chitosan-PLGA nanoparticles allowed their greater association with the intestinal epithelium [301]. In addition, ferulic-acid-chitosan-PLGA nanoparticles achieved a sustained release pattern of ferulic acid following oral administration [301]. Ferulic-acid-assembled zein-casein-lysine nanoparticles were claimed to be an efficient oral formulation of ferulic acid offering better intestinal permeability, low toxicity, and sustained release profile [302]. Ethyl-oleate-containing nanostructured lipid carriers have been reported to improve the oral bioavailability of ferulic acid compared with ferulic acid-conjugated solid lipid nanoparticles [303]. Ferulic-acid-assembled chitosan-tri-poly phosphate nanoparticles achieved an improvement in the pharmacokinetic profile of ferulic acid [155]. Ferulic acid-loaded chitosan nanoparticles have been found to be biocompatible, non-toxic, and target specific oral formulation [155]. Ferulic acid-chitosan nanoparticles achieved a sustained release profile and a ~ four-fold escalation of oral bioavailability of ferulic acid than free ferulic acid [155]. In addition, ferulic acid-chitosan nanoparticles showed excellent therapeutic effect in the reduction of blood glucose levels and enhancement of plasma insulin levels in diabetic rats compared to native ferulic acid [155]. Bairagi and co-workers claimed that both ferulic acid-PLGA nanoparticles and ferulic acid-PLGA nanoparticles-loaded carbopol 980 hydrogel exhibited a sustained release of ferulic acid from the respective formulations [304]. Oral administration of ferulic acid-PLGA nanosuspension was found to reciprocate hyperglycaemia more efficiently than free ferulic acid in wound-bearing diabetic rats [304]. In addition, ferulic-acid-PLGA nanosuspension treatment was also found to heal the diabetic wound [304]. Topical ferulic-acid-PLGA nanogel could significantly heal the diabetic wound without improving glycaemic status [304]. However, simultaneous treatment with oral ferulic-acid-PLGA nanosuspension and topical ferulic acid-PLGA nanogel was found to attenuate diabetic wound more effectively than that of any single treatment with either nanoformulation or free ferulic acid [304]. Thus, co-treatment of ferulic-acid-PLGA nanosuspension and ferulic acid-PLGA nanogel has been found to be an emerging approach to mitigate hyperglycaemia and diabetic wound.

### 5.25. Other Plant-Derived Antidiabetic Nanoformulations

Betulin nanoparticles prepared by antisolvent precipitation method were found to enhance oral bioavailability (~ 2.2-fold) and hypoglycaemic effect of betulin more efficiently in diabetic mice than native betulin [156]. Self-nanoemulsifying drug delivery of trans-cinnamic acid has been found to alleviate hyperglycaemia, hyperlipidaemia, and diabetes-provoked toxicity more efficiently in diabetic rats via improving oral absorption and bioavailability of trans-cinnamic acid than free drug [305]. Trigonelline nanoparticles were claimed to be an excellent nanoscale device to attenuate hyperglycaemia and diabetes-provoked oxidative stress in the liver and pancreas [306]. Crocetin, a carotenoid in saffron, have been found to alleviate hyperglycaemia and hyperglycaemia-triggered oxidative tissue injury [307]. Crocetin-loaded PLGA nanoparticles have been found to alleviate hyperglycaemia, haemoglobin glycation, and diabetic nephropathy more efficiently than free crocetin via improving oral bioavailability, stability, and tissue uptake of crocetin [161]. Crocetin-PLGA nanoparticle treatment caused a dramatic improvement in redox parameters, fibrotic markers, and inflammatory factors in the renal tissue of diabetic rats compared to native crocetin [161]. Rhein, a bioactive anthraquinone in rhubarb species, has been reported to reduce hyperglycaemia, improve glucose tolerance, protect pancreatic β cells, and inhibit diabetic complications [308,309]. Rhein-loaded liponanoparticles comprising polycaprolactone-polyethyleneimine-based cores have been found to achieve an excellent capacity of targeting and accumulation of rhein in kidneys with minimal urinary excretion to achieve better therapeutic efficacy in the management of diabetic nephropathy in mice [310]. Rhein liponanoparticles exhibited good stability in the biological environment and a sustained release profile up to 48 h without producing any toxic effect to the renal cells [310]. 14-Deoxy 11, 12-didehydro andrographolide-loaded polycaprolactone (PCL) nanoparticles exhibited more stability, superior cellular uptake, and prolonged release profile to attain a dramatic improvement in glucose uptake by skeletal muscle cells than free drug [311]. An in vitro drug release study of this nanoformulation demonstrated an initial burst release at 24 h followed by a sustained release for up to 11 days of 14-deoxy 11, 12-didehydro andrographolide [311]. Vicenin 2, naturally occurring flavonoid glycoside, is known for its potential antidiabetic effect via promoting AMPK/GLUT4-mediated glucose uptake and inhibiting α-glucosidase, protein tyrosine phosphatase 1B (PTP1B), AGEs formation, aldose reductase, inflammation, and oxidative stress [312,313]. Vicenin-gold nanoparticles have been reported to improve glucose uptake by 3T3-L1 adipocytes than free drug via improving the stability of vicenin 2 [314]. Fisetin, a dietary flavonol, is known to improve glucose homeostasis and mitigates diabetic complications [43]. Several nanoformulations, such as fisetin-PLGA-hyroxypropyl β-cyclodextrin nanoparticles, fisetin-assembled solid lipid nanoparticles, and fisetin-loaded nanoemulsion, were reported to improve oral bioavailability and gastrointestinal stability of fisetin [165,315,316]. Fisetin-PCL-PLGA-PEG-COOH nanoparticles have been claimed to be an attractive formulation with an improved pharmacokinetic profile for efficient management of hyperglycaemia and oxidative stress [317]. Astaxanthin, a naturally occurring keto-carotenoid, exhibits strong antioxidant and antidiabetic effects [318]. Astaxanthin-assembled nanostructured lipid carriers demonstrated the capability of improving the stability and enhancing the antioxidant activity of astaxanthin [168]. Astaxanthin-loaded chitosan oligosaccharides-PLGA core-shell nanoparticles have been found to improve the water solubility, stability, bioavailability, cytocompatibility, and sustained release profile of astaxanthin as compared to native astaxanthin [319]. Chitosan-oligosaccharides (chitooligosaccharides) can consequently exhibit a hypoglycaemic effect [320]. Astaxanthin-chitooligosaccharides-PLGA nanoparticles would be an efficacious antidiabetic formulation to mitigate hyperglycaemia and associated complications. Transdermal delivery of astaxanthin-α-tocopherol-κ-carrageenan nanoemulsion was found to reduce blood glucose levels, improve glucose tolerance, and accelerate wound healing in diabetic mice [321]. Lycopene is a red-colored carotenoid abundant in tomatoes and other red fruits. It possesses antidiabetic and antioxidant properties. However, stability is the most critical issue with lycopene. In order to improve its stability, Sharma and co-workers formulated lycopene-encapsulated niosomes [322]. Lycopene niosomes were found to enhance the stability and prolong the release of lycopene up to 72 h [322]. Lycopene-loaded niosomes have been observed to alleviate hyperglycaemia and dyslipidaemia more efficiently than the native lycopene in diabetic rats [322]. Bixin, a red coloured apocarotenoid, possesses hypoglycaemic activity [10]. Like lycopene, poor stability is a major limitation of bixin. Bixin-assembled solid lipid nanoparticles were reported to improve its clinical applications by enhancing the stability, localization at targeted tissues, sustained drug release by passive diffusion, and cellular uptake of bixin [323]. Bixin-loaded PCL nanofibers were found to be a biocompatible formulation and exhibited an initial burst release (30%–40%, in the first 10 h) followed by a sustained release (100% in day 14) of bixin. Bixin-PCL nanofibers exhibited the excellent ability of tissue regeneration and wound repair to promote the healing of diabetic wounds in mice [324]. Lutein, a xanthophyll carotenoid, has been reported to be useful in preventing hyperglycaemia-induced oxidative stress, inflammation, and cataract development without affecting glycaemic status [325,326]. Lutein nanoformulations indeed corrected the pharmaceutical incompetence of lutein [327,328]. Lutein-PVA nanoparticles alleviated hyperglycaemia, hyperlipidaemia, and hepato-renal oxidative stress in diabetic rats more efficiently at much lower doses than the native lutein [329]. In addition, free lutein failed to impress with its hypoglycaemic effect [329]. Fucoxanthin, a marine xanthophyll, exhibits antidiabetic effect through promoting insulin responsiveness and glucose uptake by skeletal muscle via triggering PPAR-γ co-activator 1-α (PGC-1α) and Akt/GLUT4 activation [330]. Simultaneously, it prevents obesity and diabetes-related disorders [331]. Fucoxanthin-casein-chitosan, fucoxanthin-zein-caseinate, fucoxanthin-pinolenic acid, fucoxanthin-chitosan-glycolipid nanoformulations were reported to enhance stability, safety, bioavailability, and therapeutic efficacy of fucoxanthin [331,332,333,334]. However, the exact effect of fucoxanthin nanoparticles on diabetic animals is yet to be explored. 16-hydroxycleroda-3,13-dine-16,15-olide, a naturally occurring DPP-4 inhibitor from *Polyalthia longifolia*, exhibits hypoglycaemic activity. 16-Hydroxycleroda-3,13-dine-16,15-olide in mesoporous silica nanoparticles reciprocated hyperglycaemia, insulin resistance, dyslipidaemia and obesity in diabetic mice more efficiently than native drug [174]. γ-Oryzanol, a mixture of phytosterol ferulates and triterpene alcohol extracted from rice bran oil, is a PPAR-γ agonist and can promote glucose utilization via IRS-1/PI3K/Akt/GLUT4 activation [335]. γ-Oryzanol-loaded PLGA nanoparticles have been found to improve absorption efficiency and therapeutic efficacy of γ-oryzanol [336]. Nano-γ-oryzanol effectively ameliorated blood glucose and lipid metabolism at a 1000-fold lower dose than native γ-oryzanol in type 2 diabetic mice [336]. In addition, a noticeable impact has been achieved through its dosing once every two weeks. γ-Oryzanol nanoparticles were found to exhibit a significant decrease in toxicosis in hypothalamus, pancreatic islets, liver, and adipose tissue [337]. Nicotinamide has been reported to stimulate pancreatic β cells to produce insulin [337]. Nicotinamide-functionalized multi-walled carbon nanotubes have been found to restore cell viability of insulin-producing β cells and increase insulin production more significantly than free nicotinamide via triggering macrophage migration inhibitory factor (MIF) pathway [337]. Propanoic acid 2-(3-acetoxy-4,4,14-trimethylandrost-8-en-17-yl), a antidiabetic lead in *Cassia auriculata* flower, showed significant antidiabetic activity [338]. However, propanoic acid 2-(3-acetoxy-4,4,14-trimethylandrost-8-en-17-yl)-fabricated gold nanoparticle treatment for four weeks has been found to reduce hyperglycaemia, suppress hyperlipidaemia, improve insulin level, and inhibit loss of body weight more efficiently than free drug in type 1 diabetic rats [338,339]. In addition, propanoic acid 2-(3-acetoxy-4,4,14-trimethylandrost-8-en-17-yl)-gold nanoparticles significantly attenuated PTP1B activity in vitro at a much lower concentration [340]. In another report, propanoic-acid-functionalized gold nanoparticles have been found to elicit antidiabetic potential in vitro via promoting glucose uptake by skeletal muscle, suppressing PTP1B, and inhibiting glucose absorption through α-glucosidase and α-glucosidase inhibition [339]. Escin, a bioactive mixture of saponins in horse chestnut seeds, is known for its hypoglycaemic and anti-inflammatory effects [340,341]. To improve therapeutic efficacy and biocompatibility, Shamprasad and co-workers formulated escin-fabricated gold nanoparticles via natural sunlight-mediated synthesis [342]. Escin-gold nanoparticles have been found to possess better pharmacological efficacy in improving glucose uptake by skeletal muscle and promoting radical scavenging effect in vitro than native escin [342]. Guavanoic acid, an antidiabetic lead from *Psidium guajava* leaves, has been formulated as guavanoic-acid-gold nanoparticles and separated by the classical chromatographic methods [343]. Guavanoic acid-gold nanoparticles were found to be a stable formulation with better PTP1B inhibitory capacity (IC_50_ ~ 2.2 µM) than RK-682 (IC_50_ ~ 5 µM), a well-known phosphatase inhibitor [343]. Docosahexaenoic acid, a long-chain polyunsaturated fatty acid, possesses antidiabetic effect via multiple mechanisms [179]. However, instability, poor pharmacokinetic profile, and age-related differential responses compromise its therapeutic utility [344]. Thus, suitable formulations of docosahexaenoic acid need to be developed to enhance its therapeutic efficacy. Gum-arabic-capped docosahexaenoic-acid-loaded zinc oxide nanoparticles have been found to be a reliable oral formulation to treat diabetes. Docosahexaenoic-acid-zinc-oxide nanoparticles could more efficiently attenuate diabetes than free docosahexaenoic acid evidenced from superior control against hyperglycaemia, insulin resistance, hyperlipidaemia, hypoinsulinaemia, and oxidative stress in diabetic rats [345]. Similarly, Docosahexaenoic-acid-silver nanoparticles could more efficiently reduce hyperglycaemia, insulin resistance, hyperlipidaemia, hypoinsulinaemia, oxidative stress, and endothelial dysfunction than native docosahexaenoic acid [346].

### 5.26. Green-Synthesized Nanoformulations as Antidiabetic Phytotherapeuticals

The global interest toward natural medicines is continuously increasing, especially to treat chronic diseases. Natural medicines include nature-derived products with nominal processing [347]. Nature-derived therapeutic agents have been regarded as eco-friendly, safe, and cost-effective healthcare agents [348]. Herbal drugs occupy the principal share in nature-derived medicines. Herbal medicines have a long and successful history in the management of diabetes and diabetic complications [349,350]. Several medicinal plants, such as *Gymnema sylvestre*, *Momordica charantia*, *Azadirachta indica, Trigonella foenum gracecum, Tinospora cordifolia, Inula racemosa, Allium sativum, Eugenia jambolana, Syzygium cumini, Pterocarpus marsupium, Emblica officinalis, Asparagus racemosus, Boerhavia diffusa* etc., have been reported to be clinically useful in the management of diabetes [349]. Green synthesis of herbal product-assembled polymeric or metallic nanoparticles has been found to achieve better therapeutic output in the management of diabetes than native crude products [351,352,353,354]. Phyto-nanotherapy offers better biopharmaceutical attributes and is revealed to be clinically equivalent to many commercially available antidiabetic drugs [353]. In addition, plant-metal nanoparticles can achieve unique therapeutic properties via a synergistic effect [353]. Green synthesis of gold, silver, and zinc oxide nanoformulations of herbal drugs has got considerable attention through improving the stability, pharmacokinetic attributes, and biopharmaceutical properties of comprising phytochemicals to achieve better therapeutic efficacy in the management of diabetes [353]. Several phyto-nanoformulations developed in recent years have been claimed to be effective in alleviating diabetes (Supplementary Table S1). However, a substantial amount of research is required in developing novel antidiabetic phyto-nanoformulation(s) to be clinically useful against diabetes.

## 6. Present Scenario and Future Perspective

Nanotechnology has appreciably emerged in the medicinal field in recent years. Patient compliance has been regarded as an important aspect in the treatment of diabetes, where prolonged or continuous treatment is required. Nanoformulations have been found to improve patient compliance by offering various routes of administration, regulating release, improving biological stability, achieving target specificity, and reducing toxicity. Thus, interest in developing nanoformulations against diabetes has been increasing dramatically, as evidenced by the published documents in the last 20 years (Supplementary Figure S1). Similarly, natural-product-based different nanoformulations and natural-product-based antidiabetic nanoformulations have fetched the interest of the researchers over the years (Supplementary Figure S1). Considering these, it would be said that there would have the possibility of developing suitable nanoformulations to attenuate diabetes and associated complications using naturally occurring antidiabetic agents or crude herbal products.

Several naturally occurring agents with well-established antidiabetic potential have been nanoformulated to improve their pharmacokinetic and therapeutic efficacy in the management of diabetes mellitus. However, the majority of these studies have been constrained due to inadequate data and lack of long term experimental statistics, particularly, regarding the prolonged stability profiling, long-term therapeutic effectiveness, and toxicological characteristics of the developed antidiabetic nanoformulations of plant-derived molecules. Thus, most of the findings are confined within the laboratory scale. Thus, significant attention must be paid to resolve this issue. On the other hand, several nanoformulations of plant-derived molecules also possessing antidiabetic effects have also been developed and reported to be effective against other diseases etiologically similar or different from diabetes [111,278,355,356,357,358,359]. Thus, dose manipulation of these nanoformulations would have the prospects in the therapeutic management of diabetes. Presently, around 51 FDA-approved nanoformulations are applied clinically as medicines and all these nanoformulations have been initially designed to improve physicochemical, pharmaceutical, and pharmacokinetic attributes of comprising drug molecules [360]. Considering these, it would be expected that the nanoformulations of naturally occurring hypoglycaemic agents will offer huge prospects through improving patient compliance, ensuring cost-effectiveness, and reducing toxicity in diabetes therapy in the future.

## 7. Interpretation and Conclusion

In the case of chronic metabolic syndrome like diabetes, long-term treatment is required. Thus, patient compliance is the most needed criterion in developing the formulation of pharmacotherapeutic agents for diabetes management. In this regard, oral formulation is mostly preferred. Plant-derived antidiabetic molecules offer excellent prospects to attenuate diabetes and associated complications. However, the poor biopharmaceutical and pharmacokinetic profiles of phytochemicals largely limit their therapeutic efficacy. Over the years, several antidiabetic nanoformulations of plant-derived molecules, such as polymeric nanoparticles, nano-emulsion, nanocarrier-assembled nanoparticles, nanoliposomes, solid lipid nanoparticles, nanostructured lipid carriers, nanomicelles, solid dispersions, and nanocrystals have been developed. 

PLGA has been claimed as the potential nanocarrier for the oral delivery of most of the plant-based compounds, including curcumin, resveratrol, quercetin, silymarin, pelargonidin, thromboquinone, ferulic acid, crocetin, and γ-oryzanol [193,221,272,273,292,300]. Although PLGA-loaded nanoformulations have been claimed to improve oral bioavailability and antidiabetic efficacy of aforementioned plant-derived small molecules, PLGA is very much susceptible to the hydrolytic degradation in gastrointestinal environment. In this regard, the PLA/PGA ratio in PLGA is a critical factor and the effects of different grades of PLGA as nanocarriers need to be specifically addressed. Chitosan has been regarded as a suitable nanocarrier for the oral delivery of curcumin, naringenin, quercetin, gymnemic acid, rosmarinic acid, scutellarin, silybin, and others, which improves compliance and pharmacokinetic attributes of the aforementioned antidiabetic phytochemicals [200,220,224,240,247,268]. Pluronic has also been found to be another suitable nanocarrier in developing curcumin, apigenin, baicalin, stevioside, silymarin, silybin, and thymoquinone nanoformulations [121,138,148,196,232,269,295,297].

Fabrication of nanocarriers can offer better drug loading capacity, gradual release, low toxicity, and antidiabetic efficacy of the formulations [192]. Development of different block polymers such as nanocarriers using PLGA, PEG, PCL, poly(ε-caprolactone), polyacrylamide, chitosan, pluronic etc. as di- or tri-blocks has also been shown to improve the therapeutic efficacy of phytochemicals as antidiabetic agents with better pharmacokinetic attributes. PLGA-PVA, Poly(ε-caprolactone)-PLGA, pluronic-PLGA, PLGA-PEG, chitosan-PLGA, chitosan-gum arabic, chitosan-alginate, PCL-PLGA-PEG-COOH, and chitooligosaccharides-PLGA, polycaprolactone-polyethyleneimine, polyacrylamide-chitosan-PLGA, chitooligosaccharides-PLGA, and PEG-block-[poly-(ethylenediaminel- glutamate)-graft-poly-(ε-benzyloxy-carbonyl-l-lysine)] have been revealed to be attractive nanocarriers to improve pharmacokinetic profile and antidiabetic efficacy of curcumin, luteolin, silybin, thymoquinone, ferulic acid, fisetin, and astaxanthin, respectively [195,223,237,244,269,317,321]. In addition, block polymer can improve the target specificity of the nanoformulations [361]. Chemical modifications of polymeric nanocarriers can also improve the pharmacokinetic profile and compliance over unmodified polymers. Galactosylation of PLGA can yield superior nanocarrier in terms of improvement in transepithelial transport, oral bioavailability, and therapeutic efficacy of resveratrol than the unmodified PLGA [214]. Similarly, succinylated chitosan-alginate and o-hexadecyl-dextran blocks have been regarded as suitable nanocarriers for the oral delivery of naturally occurring antidiabetic molecules [224,256].

In conclusion, the nanoscale formulations of plant-derived antidiabetic molecules have been found to improve the compliance and clinical efficacy by overturning the pharmacokinetic and biopharmaceutical obstacles associated with them. Thus, the development of nanoformulations could be anticipated as a potential solution to achieve the best clinical output of plant-derived antidiabetic molecules. However, more research is required to deliver clinically effective therapeutic nanoformulations of plant-derived antidiabetic molecules to control diabetes and associated complications.

## Figures and Tables

**Table 1:** Pharmaceutical limitations of plant-derived antidiabetic molecules.

S No.	Compounds	Pharmaceutical Limitations	References
1	Curcumin	Water solubility ~ 8 mg/L, poor chemical stability, low penetrability, poor absorption, rapid metabolism, high faecal excretion, elimination half-life ~ 2 h.	[112,113,114]
2	Resveratrol	Water solubility ~ 30 mg/L, rapid metabolism, rapid elimination, low plasma concentration, limited systemic distribution, oral bioavailability ~ 1–5%, poor physicochemical stability, rapid trans to cis (less active) isomerization.	[111,115,116]
3	Naringenin	Water solubility ~ 9.8 mg/L, low absorption, rapid metabolic transformation by the hepatic and gastric enzymes, oral bioavailability ~ 5%, high intestinal P-gp efflux.	[111,117,118]
4	Quercetin	Water solubility ~ 10 mg/L, poor chemicobiological stability, low absorption, fast metabolism, rapid elimination, poor oral bioavailability ~ 1%.	[119,120]
5	Apigenin	Water solubility ~ 16 mg/L, poor lipid solubility, high metabolic transformation, poor oral bioavailability, high inter-individual variability.	[121]
6	Myricitrin	Water solubility 300 mg/L, poor gastrointestinal stability, rapid conversion into poorer soluble myricetin (solubility ~ 17 mg/L) by colonic microflora, very low absorption, poor bioavailability.	[122,123]
7	Baicalin	Water solubility 91 mg/L, poor absorption, high biliary excretion, high metabolic conversion, poor bioavailability (~ 3 % in rats).	[124,125]
8	Luteolin	Water solubility 140 mg/L, low absorption, rapid first pass effect, bioavailability ~ 4 %.	[126]
9	Mangiferin	Water solubility ~ 300 mg/L, poor absorption, high first-pass property, rapid metabolism (by cytochrome P-450), high P-gp efflux, oral bioavailability ~ 1.5–5%.	[127]
10	Gymnemic acid	Poor water solubility, poor lipid solubility, very poor oral bioavailability.	[128,129]
11	Emodin	Water solubility ~ 222 mg/L, poor intestinal absorption, faster metabolism, rapid elimination, low bioavailability.	[130,131]
12	Rosmarinic acid	Poor biological stability, poor absorption, rapid metabolic transformation, poor bioavailability 0.9–1.7 %.	[132,133]
13	Berberine	Poor water solubility ~ 2.1 g/L, high P-gp efflux, low plasma concentration, rapid biotransformation, large intestinal and hepatic first-pass, poor oral bioavailability < 1%.	[134,135,136,137]
14	Stevioside	Poor intestinal absorption, low persistence, rapid metabolic degradation by human microflora, low bioavailability.	[138,139,140]
15	Asiatic acid	Poor water solubility ~ 158 mg/L (in saturated saline), rapid hepatic metabolism, poor oral bioavailability (~ 16% in rats)	[92,141]
16	Glycyrrhizin	Poor absorption, prosystemic hydrolysis by gastric fluid and by gastrointestinal flora, rapid hepatic metabolism, low oral bioavailability.	[142]
17	α-Eleostearic acid	Poor chemical stability, high metabolic conversion, low oral bioavailability.	[143,144]
18	Scutellarin	Water solubility ~ 15 mg/L, poor lipid solubility, poor membrane permeability, very low absorption, rapid metabolism, rapid faecal elimination, poor oral bioavailability (< 0.75% in dog).	[145,146]
19	Silymarin	Poor water solubility < 50 mg/L, poor intestinal permeability, rapid metabolism, rapid excretion, poor oral bioavailability.	[147,148]
20	Gallic Acid	Fast gastrointestinal absorption, fast systemic metabolism, rapid elimination, poor oral bioavailability.	[149]
21	Catechins	Poor stability, slow intestinal absorption, rapid P-gp efflux, fast metabolism, rapid clearance, poor oral bioavailability ~ 5%, poor cellular permeability	[150]
22	Pelargonidin	Low water solubility, poor stability, rapid metabolic degradation, poor bioavailability.	[151]
23	Thymoquinone	Poor aqueous solubility, high lipophilicity, slow absorption, fast metabolism, rapid elimination, low bioavailability, poor physicochemical stability.	[152,153]
24	Ferulic acid	Poor water solubility, poor gastrointestinal stability, rapid metabolism, low bioavailability ~ 3%.	[154,155]
25	Betulin	Low aqueous solubility, high permeability, low and variable bioavailability	[156,157]
26	Trans-cinnamic acid	Rapid absorption, rapid elimination, quick metabolism.	[158]
27	Trigonelline	Moderate absorption rate, fast elimination.	[159]
28	Crocetin	Water solubility ~ 1.2 mg/L, instability, rapid absorption, low oral bioavailability.	[160,161]
29	Rhein	Low hydrophilicity, aqueous solubility < 1 mg/L, low oral absorption, fast metabolic degradation, poor oral bioavailability t_1/2_ ~ 15 min.	[162,163]
30	14-Deoxy 11, 12-didehydro andrographolide	Poor aqueous solubility, rapid absorption, fast metabolism, poor oral bioavailability.	[164]
31	Fisetin	Water solubility ~ 10.5 mg/L, pro-systemic metabolism, rapid first pass metabolism, high P-gp efflux, low oral bioavailability.	[165,166]
32	Astaxanthin	High lipophilicity, poor water solubility, poor stability, low oral bioavailability.	[167,168]
33	Lycopene	Extensively isomerized after dosing, chemical instability, rapidly metabolized into polar metabolites, rapid excretion.	[169]
34	Bixin	Poor water solubility, very poor chemical stability.	[170]
35	Lutein	High lipophilicity, poor water solubility, poor physic-chemical stability, low oral bioavailability.	[171,172]
36	Fucoxanthin	Poor aqueous solubility, poor physic-chemical stability, low oral bioavailability.	[173]
37	16-Hydroxycleroda-3,13-dine-16,15-olide	Poor water solubility, low oral bioavailability.	[174]
38	γ-Oryzanol	Poor water solubility, rapid metabolism, poor oral bioavailability.	[175]
39	Escin isomers	Poor water solubility, Extensive metabolism in the gut, low bioavailability.	[176,177]
40	Docosahexaenoic acid	Poor water solubility, hydrophobic, low absorption, low bioavailability, redox instability, age-related differential responses.	[178,179]

## Supplementary Materials

The following are available online at https://www.mdpi.com/1422-0067/21/6/2217/s1, Table S1: Antidiabetic nanoformulations of crude natural products. Figure S1: Statistics of research in the field of nanotechnology-based formulation development accessed in scopus on 08.03.2020.
