# Supplementary material for: Plant-Based Antidiabetic Nanoformulations: The Emerging Paradigm for Effective Therapy
Source: Int J Mol Sci. 2020 Mar 23;21(6):2217. doi: 10.3390/ijms21062217 (PMC7139625; doi:10.3390/ijms21062217)
Supplement: Supplementary file 1 [file ijms-21-02217-s001.zip › Figure S1.pdf]

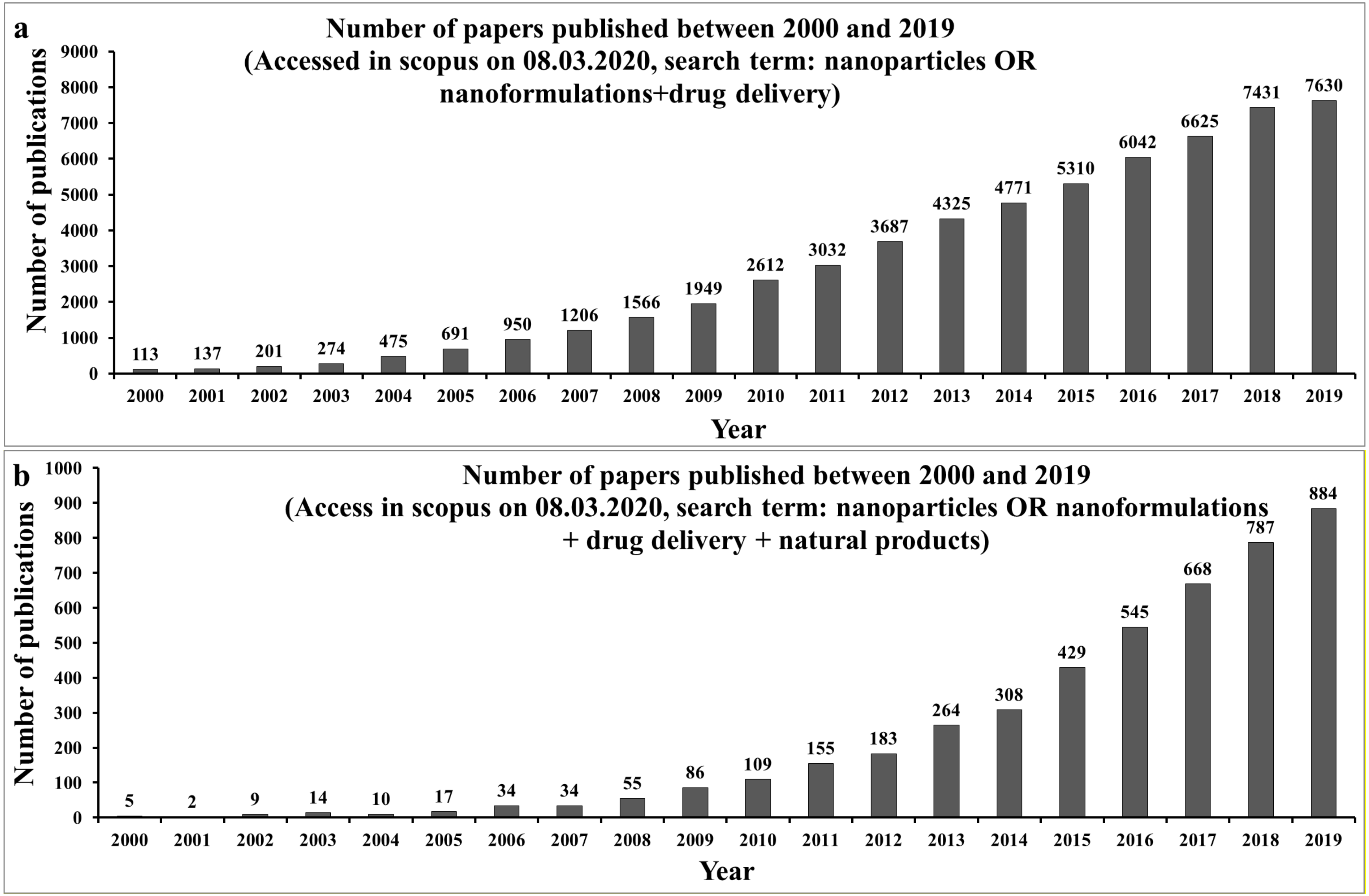
a
Number of papers published between 2000 and 2019
(Accessed in scopus on 08.03.2020, search term: nanoparticles OR nanoformulations+drug delivery)
Number of publications
0
1000
2000
3000
4000
5000
6000
7000
8000
9000
113
137
201
274
475
691
950
1206
1566
1949
2612
3032
3687
4325
4771
5310
6042
6625
7431
7630
2000
2001
2002
2003
2004
2005
2006
2007
2008
2009
2010
2011
2012
2013
2014
2015
2016
2017
2018
2019
Year
b
Number of papers published between 2000 and 2019
(Access in scopus on 08.03.2020, search term: nanoparticles OR nanoformulations + drug delivery + natural products)
Number of publications
0
100
200
300
400
500
600
700
800
900
1000
5
2
9
14
10
17
34
34
55
86
109
155
183
264
308
429
545
668
787
884
2000
2001
2002
2003
2004
2005
2006
2007
2008
2009
2010
2011
2012
2013
2014
2015
2016
2017
2018
2019
Year

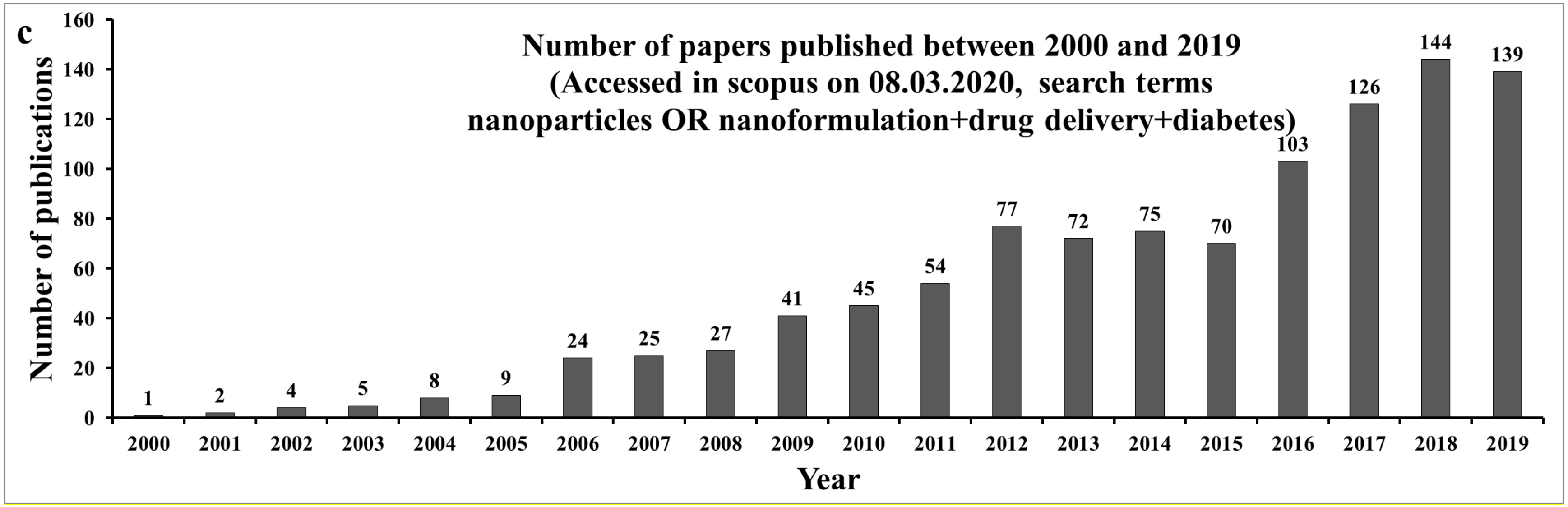

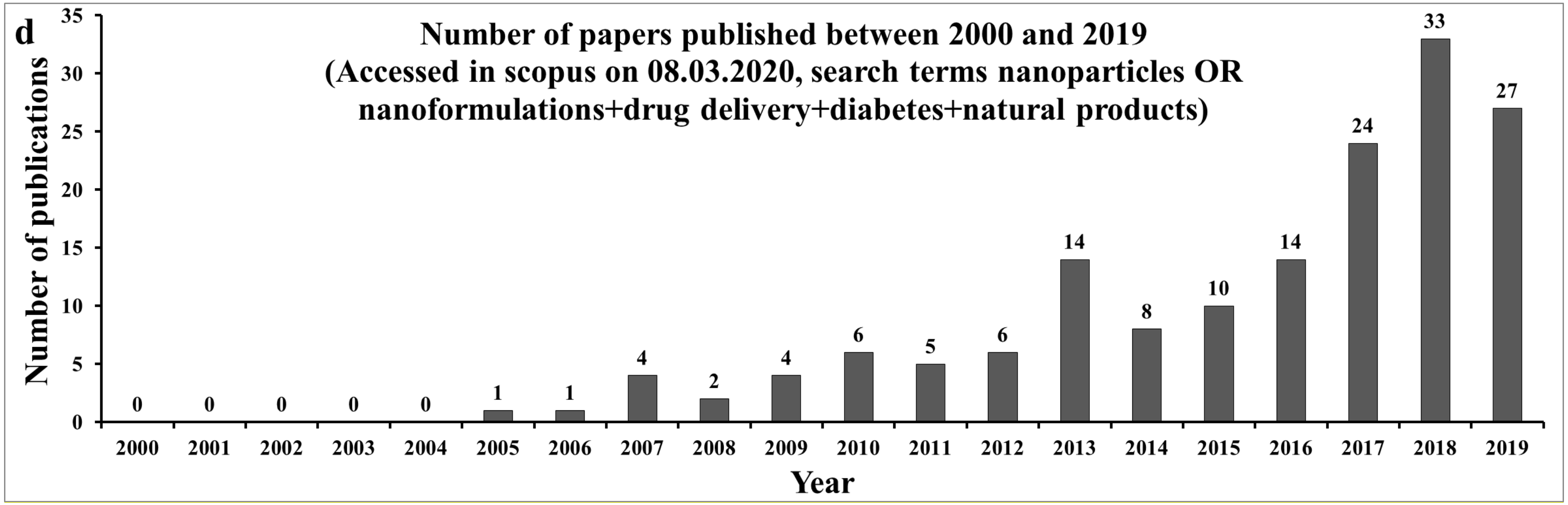

Figure S1. Statistics of research in the field of nanotechnology-based formulation development accessed in scopus on 08.03.2020. (**a**) Number of papers published between 2000 and 2019 with the search term nanoparticles OR nanoformulations+drug delivery; (**b**) Number of papers published between 2000 and 2019 with the search term nanoparticles OR nanoformulations+drug delivery+natural products; (**c**) Number of papers published between 2000 and 2019 with the search term nanoparticles OR nanoformulations+drug delivery+diabetes; (**d**) Number of papers published between 2000 and 2019 with the search term nanoparticles OR nanoformulations+drug delivery+diabetes+natural products.
