# Supplementary material for: Plant-Based Antidiabetic Nanoformulations: The Emerging Paradigm for Effective Therapy
Source: Int J Mol Sci. 2020 Mar 23;21(6):2217. doi: 10.3390/ijms21062217 (PMC7139625; doi:10.3390/ijms21062217)
Supplement: Supplementary file 1 [file ijms-21-02217-s001.zip › Table S1.pdf]

Table S1. Antidiabetic nanoformulations of crude natural products.

S. no	Plants	Nanoformulations	Models	Size (nm)	Outcomes	References
1	*Allium cepa* bulb	*A. cepa*-silver nanoparticles	*In vitro* assays	~49-73	Free radical ↓, α-glucosidase ↓, α-glucosidase ↓, cytotoxicity ↓.	[1]
2	*Ananas comosus* outer peel	*A. comosus*-silver nanoparticles	*In vitro* assays	-	Free radical ↓, reducing power ↑, NO ↓, α-glucosidase ↓.	[2]
3	*Andrographis echioides* (Syn: *Indoneesiella echioides)* whole plant	*A. echioides*-silver nanoparticles	Streptozotocin-diabetic male rats	-	Plasma glucose ↓, plasma insulin ↑, plasma hemoglobin ↑, plasma glycated hemoglobin ↓, plasma free fatty acids ↓, plasma phospholipids ↓, plasma triglycerides ↓, plasma cholesterol ↓, plasma HDL-cholesterol ↑, plasma LDL-cholesterol ↓, plasma VLDL-cholesterol ↓, serum AST ↓, serum ALT ↓, serum ALP ↓, serum GGT ↓, plasma urea ↓, plasma uric acid ↓, plasma creatinine ↓, plasma protein ↑, HK (liver and kidney) ↑, GK (liver and kidney) ↑, G6P (liver and kidney) ↑, G1,6DP (liver and kidney) ↑, G6PD (liver and kidney) ↑, lipid peroxidation (pancreas, liver, and kidney) ↓, hydroperoxides (pancreas, liver, and kidney) ↓, antioxidant enzymes (pancreas, liver, and kidney) ↑, GSH (pancreas, liver, and kidney) ↑, Vit C (pancreas, liver, and kidney) ↑, Vit E (pancreas, liver, and kidney) ↑.	[3]

4	*Andrographis paniculata* leaf & herb	*A. paniculata*-gold nanoparticles	*In vitro* assays	~57-115	α-Amylase ↓, free radicals ↓, reducing power ↓, inflammation ↓, NO ↓.	[4,5]
		A. paniculata-casein micelle nanopreparation	*In vitro* assay	~120	α-Glucosidase ↓.	
5	*Antrodia cinnamomea* tritepenoids	*A. cinnamomea* triterpenoids-silica-chitosan nanoparticles	*In vitro* assays	~79	Cellular uptake ↑, cyto-toxicity ↓, inflammation ↓, NO ↓, superoxide ↓.	[6]
			Streptozotocin-diabetic male rats		Blood glucose ↓, insulin resistance ↓, testicular SOD ↑, testicular GPx ↑, testicular CAT ↑, testicular IL-6 ↓, testicular TNF-α ↓, plasma IFN-γ ↓, plasma LH ↑, plasma testosterone ↑, plasma FSH ↑, ASK-1 ↓, c-JNK ↓, ATF-2 ↓, sperm count ↑, sperm mobility ↑, sperm abnormality ↓, sperm ROS ↓, sperm mitochondrial membrane potential ↑.	
6	*Argyreia nervosa* leaf	*A. nervosa*-silver nanoparticles	*In vitro* assays	~5-40	α-Glucosidase ↓, α-amylase ↓, free radical ↓.	[7]
7	*Avicennia officinalis* leaf	*A. officinalis*-silver nanoparticles	*In vitro assays*	~181	α-Glucosidase ↓, α-amylase ↓, free radicals ↓, inflammation ↓.	[8]
8	*Azadirachta indica* leaf	*A. indica*-zinc oxide nanoparticles	*In vitro assays*	~27-54	α-Glucosidase ↓, α-amylase ↓, free radicals ↓, H_2O_2 ↓.	[9]
9	*Barleria prionitis* leaf	*B. prionitis*-copper nanoparticles	*In vitro assays*	~11-15	α-Glucosidase ↓, α-amylase ↓.	[10]
10	*Bauhinia variegate* flower	*B.variegata*-silver nanoparticles	*In vitro assays*	~5-15	α-Amylase ↓, free radicals ↓.	[11]
11	*Botryodiplodia theobromae,* endophytic fungi	*B. theobromae*-silver nanoparticles	*In vitro assay*	~53-79	α-Amylase ↓.	[12]

12	*Breynia retusa* leaf	*B. retusa*-silver nanoparticles	*In vitro* assays	~30-40	α-Amylase ↓.	[13]
13	*Caesalpinia coriaria* leaf and bark	*C. coriaria*-silver nanoparticles	*In vitro* assays	-	Glucose uptake (yeast cells) ↑, inflammation ↓.	[14]
14	*Calophyllum tomentosum* leaf	*C. tomentosum*-silver nanoparticles	*In vitro* assays	~24	Free radicals ↓, reducing power ↑, NO ↓, inflammation ↓, lipoxygenase ↓, xanthine oxidase ↓, α-glucosidase ↓, DPP-4 ↓, α-amylase ↓.	[15]
15	*Cassia fistula* bark	*Cassia fistula*-gold nanoparticles	Streptozotocin-diabetic male rats	~55-98	Blood glucose ↓, serum triglycerides ↓, serum cholesterol ↓, serum HDL-cholesterol ↑, serum LDL-cholesterol ↓, serum VLDL-cholesterol ↓, serum AST ↓, serum ALT ↓, serum ALP ↓, serum urea ↓, serum uric acid ↓, serum creatinine ↓.	[16]
16	*Cassia grandis* fruit	*C. grandis*-Eudragit L 100 55-PEG 4000-Kollicoat nanodispersion	*In vitro* assays<br><br>Alloxan-diabetic mice	~106	Stability ↑, lipase ↓, α-glucosidase ↓, free radicals ↓, cyto-toxicity ↓.<br>Blood glucose ↓, body weight ↑, food intake ↑, water intake ↓.	[17]
17	*Catathelasma ventricosum* polysaccharides	*C. ventricosum polysaccharides*-selenium nanoparticles	Streptozotocin-diabetic mice	~50	Blood glucose ↓, body weight ↑, serum triglycerides ↓, serum cholesterol ↓, serum HDL-cholesterol ↑, serum LDL-cholesterol ↓, lipid peroxidation (liver and kidney), SOD (liver and kidney), CAT (liver and kidney), GPx (liver and kidney).	[18]
18	*Catharanthus roseus var. alba* leaf	*C. roseus*-chitosan nanoparticles	Alloxan-diabetic rats	-	Blood glucose ↓, glucose tolerance ↑, body weight ↑, SOD (plasma and liver) ↑, CAT (plasma and liver) ↑, GSH	[19]

					(plasma and liver) ↑.	
19	*Centella asiatica* leaf	*C. asiatica*-silver nanoparticles	*In vitro* assays	~30-50	Glucose uptake (yeast cells) ↑, non-enzymatic hemoglobin glycosylation ↓, hemoglobin ↑, *α*-amylase ↓, free radicals ↓, H_2O_2 ↓.	[20]
20	*Chamaecostus cuspidatus* leaf	*C. cuspidatus*-gold nanoparticles	*In vitro* assays	~50	Free radicals ↓, reducing power ↑, NO ↓, lipid peroxidation ↓.	[21]
			Streptozotocin-diabetic male rats		Blood glucose ↓, insulin ↑, glycogen ↑, body weight ↑, serum protein ↑, serum cholesterol ↓, diabetic wound ↓.	
21	*Chromolaena odorata* leaf	*C. odorata*-silver nanoparticles	*In vitro* assays	-	Glucose uptake (yeast) ↑, inflammation ↓.	[14]
22	*Cinnamomum cassia* bark	*C. cassia*-silver-gold nanoparticles	Streptozotocin-diabetic male rats	~45	Blood glucose ↓, insulin sensitivity ↑, body weight ↓.	[22]
		C. cassia-silver nanoparticles	Streptozotocin-diabetic male rats	~100	Blood glucose ↓, serum urea ↓, serum creatinine ↓, serum AST ↓, serum ALT ↓, serum ALP ↓, lipid peroxidation (kidney) ↓, GSH (kidney) ↑, body weight ↑.	[23]
23	*Cinnamomum tsoi* leaf	*C. tsoi*-silver nanoparticles	*In vitro* assays	~10-30	*α*-Glucosidase ↓, *α*-amylase ↓.	[24]
24	*Citrus limon* fruit	*C. limon*-zinc oxide nanoparticles	*In vitro* assays	~11-49	*α*-Glucosidase ↓, cyto-toxicity ↓.	[25]
25	*Cladosporium* species	*Cladosporium* species-silver nanoparticles	*In vitro* assays	~24	DPP-4 ↓, *α*-glucosidase ↓, *α*-amylase ↓, glucose uptake (3T3L1 adipocytes) ↑, antioxidant activity ↑.	[26]
26	*Clausena anisata* root	*C. anisata*-silver nanoparticles	*In vitro* assays	~33	Glucose uptake (yeast cells) ↓, glucose diffusion ↓, *α*-amylase ↓, glucose binding ↑, free radical ↓.	[27]
	Clausena anisata leaf	*C. anisata*-silver nanoparticles	*In vitro* assays	~61	Glucose uptake (yeast cells) ↓, glucose	[28]

					diffusion ↓, α-amylase ↓, glucose binding ↑, free radical ↓.	
27	*Colpomenia sinuosa,* marine algae	*C. sinuosa*-silver nanoparticles	*In vitro* assays	~54-65	α-Glucosidase ↓, α-amylase ↓.	[29]
28	*Corchorus depressus* whole plant	*C. depressus*-silver nanoparticles	*In vitro* assays	~2-10	α-Glucosidase ↓.	[30]
29	*Costus igneus* leaf	*C. igneus*-zinc oxide nanoparticles	*In vitro* assays	~27	Free radicals ↓, α-glucosidase ↓, α-amylase ↓.	[31,32]
		C. igneus-gold nanoparticles	*In vitro* assay	~50-60	Glucose concentration ↓.	
30	*Costus pictus* leaf	*C. pictus*-silver nanoparticles	*In vitro* assays	-	Glucose uptake (yeast cells) ↓, non-enzymatic hemoglobin glycosylation ↓, α-glucosidase ↓, α-amylase ↓.	[33]
31	*Costus speciosus* leaf	*C. speciosus*-loaded PLGA nanoparticles	Streptozotocin-diabetic male rats	~4-12	Sustained release ↑, therapeutic dose ↓, blood glucose ↓, serum triglycerides ↓, serum cholesterol ↓, serum HDL-cholesterol ↑, serum LDL-cholesterol ↓, serum AST ↓, serum ALT ↓, serum LDH ↓, serum ALP ↓, serum ACP ↓, hepatocellular DNA damage ↓, pancreatic insulin-I mRNA ↑, pancreatic insulin-II mRNA ↑, hepatic GLUT-2 mRNA ↑, muscular GLUT-4 mRNA ↑.	[34,35]
		C. speciosus-platinum modified titanium dioxide nanoparticles	*In vitro* assays	~10-50	Free radicals ↓, H_2O_2 ↓, reducing power ↑, α-amylase ↓.	
32	*Couroupita guianensis* flower	*C. guianensis*-coated gold nanoparticles	Streptozotocin-diabetic rats	~47	Blood glucose ↓, glycated haemoglobin ↓, serum insulin ↑, serum triglycerides ↓, serum cholesterol ↓, serum HDL-	[36]

					cholesterol ↑, serum AST ↓, serum ALT ↓, serum ALP ↓, serum urea ↓, serum creatinine ↓, serum bilirubin ↓, serum protein ↑, SOD (liver) ↑, CAT (liver) ↑, GPx (liver) ↑, lipid peroxidation (liver) ↓.	
33	*Curcuma longa* leaf	*C. longa*-silver nanoparticles	*In vitro* assays	~90-111	Free radicals ↓, reducing power ↑, α-amylase ↓, NO ↓.	[37]
34	*Cymbopogon citratus* leaf	*C. citratus*-silver nanoparticles	*In vitro* assays	~75-138	α-Amylase ↓, glucose diffusion ↓.	[38]
35	*Dioscorea bulbifera* tuber	*D. bulbifera*-copper nanoparticles	*In vitro* assays	~12-16	Free radicals ↓, α-glucosidase ↓, α-amylase ↓, NO ↓, superoxide ↓.	[39]
36	*Diospyros melanoxylon* leaf	*D. melanoxylon*-PLGA nanoparticles	*In vitro* assays	~366	α-Glucosidase ↓, α-amylase ↓.	[40]
37	*Dunaliella bardawil* biomass	*Dunaliella bardawil*-encapsulated keratin nanoparticles	*In vitro* (3T3-L1 adipocytes), *in silico* assays	~159	Stability ↑, sustained release of *D. bardawil* biomass ↑, glucose uptake ↑, PTP1B ↓, AMPK ↑, GLUT-4 translocation ↑, cytotoxicity ↓.	[41]
38	*Enhalus acoroides*, sea grass	*E. acoroides*-silver nanoparticles	*In vitro* assay	~2-100	α-Glucosidase ↓.	[42]
39	*Eysenhardtia polystachya* bark	*E. polystachya*-silver nanoparticles	*In vitro* assays<br><br><br>Glucose-fed zebrafish	~10-100	Stability ↑, sustained release of *E. polystachya* ↑, β-cell survival ↑, Insulin secretion ↑, cyto-toxicity ↓.<br>Glucose ↓, triglycerides ↓, cholesterol ↓.	[43]
40	*Gnidia glauca* flower, leaf, stem	*G. glauca* flower-copper nanoparticles<br>*G. glauca* leaf-copper nanoparticles<br>*G. glauca* stem-copper nanoparticles	*In vitro assays*	~5<br>~70-93<br>-	α-Glucosidase ↓, α-amylase ↓.	[44]

41	*Gymnema sylvestre* leaf	*G. sylvestre*-chitosan nanoparticles	Streptozotocin-diabetic male rats	~50-120	Blood glucose ↓, glyoxylated haemoglobin ↓, AST ↓, ALT ↓, ALP ↓, GSH (liver & kidney) ↑, SOD (liver & kidney) ↑, CAT (liver & kidney) ↑, lipid peroxidation (liver & kidney) ↓.	[45-48]
		G. sylvestre-gold nanoparticles	Alloxan-diabetic mice	~50	Blood glucose ↓, plasma insulin ↑, plasma glycated haemoglobin ↓, plasma haemoglobin ↑, plasma triglycerides ↓, plasma cholesterol ↓, plasma HDL-cholesterol ↑, plasma LDL-cholesterol ↓, plasma TNF-α ↓, plasma IL-1β ↓, plasma IL-6 ↓.	
		G. sylvestre-chitosan reduced gold nanoparticles	High fat diet + low dose streptozotocin-diabetic rats	~14	Hyperglycemia ↓, hyperlipedimia ↓, vascular tissue damage ↓, GLUT-4 ↑, PPAR-γ ↑.	
		G. sylvestre-silver nanoparticles	Streptozotocin-diabetic male rats	-	Blood glucose ↓, serum cholesterol ↓, serum triglycerides ↓, serum LDL-cholesterol ↓, serum VLDL-cholesterol ↓.	
42	*Halymenia poryphyroides* algae	*H. poryphyroides*-silver nanoparticles	*In vitro* assays	~34-80	α-Glucosidase ↓, α-amylase ↓.	[49]
43	*Helianthus tuberosus* tuber	*H. tuberosus*-gold nanoparticles	Glucose-fed rats	~10	Glucose tolerance ↑, blood glucose ↓.	[50]
44	*Heritiera fomes* bark	*H. fomes*-silver nanoparticles<br>*H. fomes*-zinc oxide nanoparticles	*In vitro assays*	~400 (WR)<br>~40-50	α-Amylase ↓, free radical ↓, inflammation ↓.	[51]
45	*Hibiscus rosa-sinensis* leaf	*H. rosa-sinensis*-zinc oxide nanoparticles	*In vitro assays*	~27-54	α-Glucosidase ↓, α-amylase ↓, free radicals ↓, H_2O_2 ↓.	[9]
46	*Hibiscus subdariffa* leaf	*Hibiscus subdariffa*-zinc oxide nanoparticles	Streptozotocin-diabetic male rats	~16-60	Blood glucose ↓, pancreatic TNF-α protein and gene ↓, pancreatic IL-6	[52]

					protein and gene ↓, pancreatic IL-1β protein and gene ↓, pancreatic IL-4 protein and gene ↑, pancreatic IL-10 protein and gene ↑, pancreatic IR A mRNA ↑, pancreatic PKLR mRNA ↓, pancreatic GLUT-2 ↑, pancreatic GK mRNA ↑,	
47	*Holoptelea integrifolia* leaf	*H. integrifolia*-silver nanoparticles	*In vitro* assays	~32-38	Free radical ↓, metal chelating ↑, NO ↓, inflammation ↓, α-amylase ↓.	[53]
48	*Ipomoea batatas* peels Korean red skin sweet potato variety (IB1), Korean pumpkin sweet potato variety (IB2)	IB1-silver nanoparticles and IB2-silver nanoparticles	*In vitro* assays	-	Free radical ↓, α-glucosidase ↓ (IC50 = 0.36 and 0.77 μg/ml for IB1-silver and IB2-silver nanoparticles, respectively), cyto-toxicity ↓.	[54]
49	*Lawsonia inermis* leaf	*L. inermis*-silver nanoparticles<br>*L. inermis*- cerium oxide nanoparticles	Streptozotocin-diabetic male rats	~15<br>~18	Blood glucose ↓, serum insulin ↑, serum triglycerides ↓, serum cholesterol ↓, serum HDL-cholesterol ↑, serum LDL-cholesterol ↓, serum VLDL-cholesterol ↓. (silver nanoparticles effect > cerium oxide nanoparticles effect)	[55]
50	*Lentinus tuber-regium*, musroom	*L. tuber-regium*-silver nanoparticles	*In vitro assays*	~5-35	α-Amylase ↓.	[56]
51	*Litchi chinensis* peel	*L. chinensis*-copper nanoparticles	*In vitro assays*	~10-100	α-Glucosidase ↓, α-amylase ↓.	[10]
52	*Lonicera japonica* leaf	*L. japonica*-silver nanoparticles	*In vitro* assays	~20-60	Free radicals ↓, α-glucosidase ↓, α-amylase ↓.	[57]
53	*Manilkara zapota* leaf and stem	*M. zapota*-silver nanoparticles	*In vitro* assays	-	Free radicals ↓, reducing power ↑, α-amylase ↓.	[58]

54	*Marsilea quadrifolia*, water fern	*M. quadrifolia*-gold nanoparticles	*In vitro* assays	~14	Glucose uptake (3T3L1 adipocytes) ↑, cyto-toxicity (3T3L1 adipocytes) low.	[59]
55	*Momordica charantia* fruit	*M. charantia*-silver nanoparticles	Streptozotocin-diabetic rats	~100	Blood glucose ↓, insulin ↑.	[60,61]
		M. charantia-zinc oxide nanoparticles	Streptozotocin-diabetic rats	~25	Blood glucose ↓, insulin ↑.	
56	*Moricandia nitens* whole plant	*M. nitens*-gold nanoparticles	*In vitro* assay	~5-30	α-Glucosidase ↓.	[62]
57	*Moringa oleifera* leaf	*M. oleifera*-zinc oxide nanoparticles	*In vitro assays*	~27-54	α-Glucosidase ↓, α-amylase ↓, free radicals ↓, H_2O_2 ↓.	[9]
58	*Morus nigra* fruit	*Morus nigra*- cerium oxide nanoparticles	*In vitro* assays	~7.5	Glucose uptake (L6 skeletal muscle cells) ↑, cyto-toxicity ↓.	[63]
59	*Morus alba* leaf + *Pueraria Lobata*	Selenium-layered *M.alba -P. Lobata*-PLGA-PEG-DOTAP nanoparticles	*In vitro* assays<br>Sprague-Dawley rats<br>Goto-Kakizaki diabetic rats	~120	Stability ↑, sustained release ↑, cellular uptake ↑, glucose uptake (adipocytes) ↑.<br>Bioavailability ↑, blood glucose ↓.<br>Blood glucose ↓, β-cells ↑, blood ROS ↓, blood lipid peroxidation ↓, blood SOD ↑, blood GPx ↑, blood GSH ↑.	[64]
60	*Murraya koenigii* leaf	*M.* koenigii-zinc oxide nanoparticles	*In vitro assays*	~27-54	α-Glucosidase ↓, α-amylase ↓, free radicals ↓, H_2O_2 ↓.	[9]
61	*Nasturtium officinale* leaf	*N. officinale*-zinc oxide nanoparticles	Alloxan-diabetic rats	~14	Stability ↑, blood glucose ↓, insulin ↑, triglycerides ↓, cholesterol ↓, HDL-cholesterol ↑.	[65]
62	*Nigella Sativa* seed	*N. Sativa*-PLGA nanoparticles	High fat diet + low dose streptozotocin-diabetic rats	~50-94	Ischemia-modified albumin ↓, lipid peroxidation ↓ (insignificant).	[66]
63	*Nilgirianthus ciliates* fresh plant	*N. ciliates*-loaded gelatin nanoparticles	*In vitro* assays	~116-160	Stability ↑, cyto-toxicity ↓, glucose uptake (L6 skeletal muscle cells) ↑,	[67]

					adipogenesis (3T3-L1 adipocytes) ↓, lipid accumulation (3T3-L1 adipocytes) ↓.	
64	*Ocimum basilicum* leaf	*O. basilicum*-silver nanoparticles	*In vitro* assays	~100	*α*-Glucosidase ↓, *α*-amylase ↓.	[68,69]
		O. basilicum-silver-gold nanoparticles	*In vitro* assays	~3-25	*α*-Glucosidase ↓, *α*-amylase ↓.	
65	*Ocimum sanctum* leaf	*O. sanctum*-silver nanoparticles	*In vitro* assays	~100	*α*-Glucosidase ↓, *α*-amylase ↓.	[68]
67	*Padina boergesenii,* brown seaweed	*P.boergesenii*-gold nanoparticles	*In vitro* assay	~2-100	*α*-Glucosidase ↓.	[70]
68	*Parmelia perlata* lichen	*P. perlata*-silver nanoparticles	*In vitro* assays	~10-100	*α*-Amylase ↓, antioxidant activity ↑, reducing power ↑, H_2O_2 ↓.	[71]
69	*Phyllanthus emblica* fruit	*P. emblica*-PLGA nanoparticles	Streptozotocin-diabetic rats	-	Blood glucose ↓, hepatic DNA damage ↓, hepatocellular GK mRNA ↑, hepatic hepatic G6Pase mRNA ↓, GLUT-2 mRNA ↑, muscular GLUT-4 mRNA ↑, GPx ↑.	[72]
70	*Pisum sativum* outer peel	*P. sativum*-silver nanoparticles	*In vitro* assays	~10-25	*α*-Glucosidase ↓, free radical ↓.	[73]
71	*Platanus orientalis* leaf	*P. orientalis*-copper nanoparticles	*In vitro assays*	~90-200	*α*-Glucosidase ↓, *α*-amylase ↓.	[10]
72	*Pleurotus giganteus,* mushroom	*P. giganteus*-silver nanoparticles	*In vitro assays*	~2-20	*α*-Amylase ↓.	[74]
73	*Plicosepalus acacia* whole plant	*P. acacia*-solid lipid nanoparticles	High fat diet + low dose streptozotocin-diabetic rats	~22-70	Blood glucose ↓, glycated haemoglobin ↓, insulin resistance ↓, insulin sensitivity ↑, lipid peroxidation ↓, GSH ↑, SOD ↑, CAT ↑.	[75]
74	*Plicosepalus curviflorus* whole plant	*P. curviflorus*-solid lipid nanoparticles	High fat diet + low dose streptozotocin-	~22-70	Blood glucose ↓, glycated haemoglobin ↓, insulin resistance ↓, insulin sensitivity	[75]

			diabetic rats		↑, lipid peroxidation ↓, GSH ↑, SOD ↑, CAT ↑.	
75	*Plumbago zeylanica* leaf	*P. zeylanica* leaf-copper nanoparticles	*In vitro* assays	~1-5	α-Glucosidase ↓, α-amylase ↓.	[44]
77	*Pouteria sapota* leaf	*P. sapota*-silver nanoparticles	*In vitro* assay	~20-110	Glucose uptake ↑, non-enzymatic hemoglobin glycosylation ↓, α-glucosidase ↓.	[76]
			Streptozotocin-diabetic rats		Blood glucose ↓, serum insulin ↑, serum AST ↓, serum ALT ↓, serum ALP ↓, serum urea ↓, serum uric acid ↓, serum creatinine ↓, serum bilirubin ↓, serum protein ↑, SOD (liver) ↑, CAT (liver) ↑, lipid peroxidation (liver) ↓.	
78	*Psoralea corylifolia* seed	*P. corylifolia*-silver nanoparticles	*In vitro* assay	~15-25	PTP1B ↓.	[77]
79	*Pterocarpus marsupium* bark and wood	*P. marsupium*-silver nanoparticles	*In vitro* assay	~148.5	α-glucosidase ↓.	[78]
80	*Pterocarpus santalinus* wood	*P. santalinus*-zinc oxide nanoparticles	*In vitro* assays	~20	α-Glucosidase ↓, α-amylase ↓.	[79]
81	*Punica granatum* leaf	*P. granatum*-silver nanoparticles	*In vitro* assays	~35-60	Free radicals ↓, α-glucosidase ↓, α-amylase ↓.	[80]
83	*Rumex hymenosepalus* root	*R. hymenosepalus*-silver nanoparticles	Streptozotocin-diabetic rats	~6-12	Blood glucose ↓, glucose tolerance ↑.	[81]
84	*Sambucus nigra* fruit	*S. nigra*-gold nanoparticles	Streptozotocin-diabetic rats	~4-26	Blood glucose ↓ (insignificant), serum insulin ↑ (insignificant), serum cholesterol ↓ (insignificant), serum AST ↓, lipid peroxidation (liver) ↓, GSH/GSSG (blood) ↑, COX-2 (liver) ↓, Pro-MMP-2 (liver) ↓, Kupffer cells ↓.	[82]

85	*Saraca asoca* leaf	*S. asoca*-gold nanoparticles<br>*S. asoca*-silver nanoparticles	*In vitro* assay	~24<br>~36	α-Amylase ↓ (IC_{50}: 1.5 mM)<br>α-Amylase ↓ (IC_{50}: 0.35 mM)	[83]
86	*Sargassum swartzii*	*S. swartzii*-gold nanoparticles	Alloxan-diabetic rats	~37	Blood glucose ↓, glycated haemoglobin ↓, haemoglobin ↑, plasma insulin ↑, serum triglycerides ↓, serum cholesterol ↓, serum HDL-cholesterol ↑, serum LDL-cholesterol ↓, serum AST ↓, serum ALT ↓, serum ALP ↓, serum creatinine ↓, serum IL-6 ↓, serum TNF-α ↓, serum high-sensitive CRP ↓.	[84]
87	*Sesamum indicum* seed	*S. indicum*-iron nanoparticles	*In vitro* assay	~99	α-Amylase ↓.	[85]
88	*Silybum marianum* seed	*S. marianum*-zinc oxide nanoparticles	Alloxan-diabetic rats	~25	Blood glucose ↓, serum insulin ↑, serum triglycerides ↓, serum cholesterol ↓, serum HDL-cholesterol ↑.	[86]
89	*Solanum nigrum* leaf	*S. nigrum*-silver nanoparticles	Alloxan-diabetic rats	~4-25	Blood glucose ↓, glucose tolerance ↑, body weight ↑, serum triglycerides ↓, serum cholesterol ↓.	[87]
90	*Sonneratia apetala* leaf	*S. apetala*-silver nanoparticles<br>*S. apetala*-zinc oxide nanoparticles	*In vitro assays*	~20-100<br>~400-500	α-Amylase ↓, free radical ↓, inflammation ↓.	[51]
91	*Sphaeranthus Amaranthoides* whole plant	*S. amaranthoides*-silver nanoparticles	*In vitro* assay	~50-150	α-Amylase ↓.	[88]
92	*Stevia rebaudiana* leaf	*S. rebaudiana*-tripolyphosphate-chitosan nanoparticles	Streptozotocin-diabetic rats	~327	Blood glucose ↓, serum insulin ↑, serum AST ↓, serum ALT ↓, serum ALP ↓, SOD (liver and kidney) ↑, CAT (liver and kidney) ↑, GSH (liver and kidney) ↑, lipid peroxidation (liver and kidney) ↓.	[89,90]
		S. rebaudiana-titanium oxide nanoparticles	Alloxan-diabetic rats	~4-100	Blood glucose ↓, glycated haemoglobin ↓, serum insulin ↑, serum triglycerides	

					↓, serum cholesterol ↓, body weight ↑.	
93	*Stoechospermum marginatum* seaweed	*S. marginatum*-silver nanoparticles	*In vitro* assays	-	α-Amylase ↓, cytotoxicity ↓.	[91]
94	*Syzygium cumini* seed	*S. cumini*-silver nanoparticles	*In vitro* assays<br>Glucose-stressed H9C2 cardiac cells	~40-100	Free radicals ↓.<br>Nuclear abnormalities ↓, cell size ↓, lipid peroxidation ↓.	[92,93]
		S. cumini polymeric nanoparticles	Streptozotocin-diabetic rats inoculated with *Candida albicans*.	-	Adenosine deaminase ↓, 5′-nucleotidase activity ↓, AMP hydrohysis ↓, NTPDase activity ↓, ADP ↓.	
95	*Syzygium jambolanum* seed	*S. jambolanum*-PLGA nanoparticles	*In vitro* assays	~122	Cyto-toxicity ↓, glucose uptake (L6 skeletal muscle cells) ↑, ROS ↓, NF-κB ↓, iNOS ↓, GK mRNA ↑, GLUT-4 protein and gene ↑.	[94]
			Arsenic-hyperglycemic mice		Blood glucose ↓, glycated haemoglobin ↓.	
96	*Talinum portulacifolium* whole plant	*T. portulacifolium* solid lipid nanoparticles	High fat diet + streptozotocin-diabetic rats	~260	Blood glucose ↓, serum triglycerides ↓, serum cholesterol ↓, serum HDL-cholesterol ↑, serum LDL-cholesterol ↓, serum AST ↓, serum ALT ↓, serum CAT ↑, serum GSH ↑, serum lipid peroxide ↓.	[95]
97	*Tamarindus indica* leaf	*T. indica*-zinc oxide nanoparticles	*In vitro assays*	~27-54	α-Glucosidase ↓, α-amylase ↓, free radicals ↓, H_2O_2 ↓.	[9]
98	*Tephrosia tinctoria* stem	*T. tinctoria*-silver nanoparticles	*In vitro* assays	< 100	α-Glucosidase ↓, α-amylase ↓, glucose uptake (erythrocytes) ↑, free radicals ↓.	[96]
99	*Tinospora cordifolia* stem	*T. cordifolia*-PLA-PVA nanoparticles	*In vitro* assays	~230	Sustained release ↑, α-glucosidase ↓.	[97]

100	*Trigonella foenum graecum* seed	*T. foenum graecum*-PLGA nanoparticles	Alloxan-diabetic male mice	-	Body mass ↑, pancreatic gland mass ↑, lipid peroxidation ↓, lipofuscin granules ↓.	[98,99]
		T. foenum graecum-silver-gold nanoparticles	Streptozotocin-diabetic rats	~73	Blood glucose ↓, serum ALT ↓, serum urea ↓, serum creatinine ↓.	
101	*Vaccinium arctostaphylos* fruit	*V. arctostaphylos*-zinc oxide nanoparticles	Alloxan-diabetic rats	~15	Blood glucose ↓, serum insulin ↑ (insignificant), serum triglycerides ↓ (insignificant), serum cholesterol ↓, serum HDL-cholesterol ↑.	[100]
102	*Withania coagulans* plant	*W. coagulans* antidiabetic fraction-chitosan assembled enteric-coated nanoparticles	*In vitro* assay<br>Alloxan-diabetic mice	-	Insulin secretion ↑ (MIN6 cells).<br>Blood glucose ↓, bioavailability ↑.	[101]
103	*Withania somnifera* leaf	*Withania somnifera*-platinum nanoparticles	Streptozotocin-diabetic rats	~12	Plasma glucose ↓.	[102]
104	*Withania somnifera* root	*W. somnifera*-silver nanoparticles	*In vitro* assays	~123	α-Glucosidase ↓, α-amylase ↓.	[103]
105	*Vaccinium arctostaphylos* leaf	*V. arctostaphylos*-zinc oxide nanoparticles	Alloxan-diabetic rats	~12-27	Blood glucose ↓, serum insulin ↑, serum triglycerides ↓, serum cholesterol ↓, serum HDL-cholesterol ↑.	[100,65]
106	*Xylocarpus granatum* bark	*X. granatum*-silver nanoparticles	*In vitro assays*	~99	α-Glucosidase ↓, α-glucosidase ↓, free radicals ↓, inflammation ↓.	[8]
	**Natural formulations /mixtures**					
107	Jiaosanxian (charcoal used in TCM)	Jiaosanxian-derived carbon dots	Glucose-hyperglycemic mice	~4.4-6.4	Solubility ↑, blood glucose ↓.	[104]
108	Ge-Gen-Qin-Lian-Tang decoction (polyherbal formulation in TCM)	Ge-Gen-Qin-Lian-Tang micro/nano-aggregates	*In vitro* assays	~300-1000	Baicalin cellular uptake ↑ (Caco-2 cells), β-cell proliferation ↑ (INS-1 cells), oxidative stress ↓ (streptozotocin-INS-1 cells), insulin secretion ↑ (glucose-INS-1	[105]

					cells)	
109	Jasada bhasma or Yashada bhasma (Ayurvedic bhasma)	Zinc-based nanoparticles	Streptozotocin-diabetic rats	~200-500	Oxidative stress ↓, Blood glucose ↓, glucose tolerance ↑, insulin sensitivity ↑.	[106]
110	Clinoptilolite, natural zeolite (Na, K, Al, and Si containing mineral linked through oxygen)	Nano-sized clinoptilolite	Streptozotocin-diabetic rats	~30-40	Blood glucose ↓, weight loss ↓, serum LDL-cholesterol ↓, serum lipid peroxidation ↓.	[107,108]

ACP: Acid phosphatase, ADP: adenosine diphosphate, ALP: Alkaline phosphatase, ALT: Alanine aminotrasferase, AMP: adenosine monophosphate, AMPK: 5' AMP-activated protein kinase, AST: Aspartate aminotransferase, CAT: catalase, COX: cyclooxygenase, DPP-4: dipeptidyl peptidase-4, F1,6DP: fructose 1,6-bisphosphatase, G6P: glucose 6-phosphatase, G6Pase: glucose-6-phosphatase, G6PD: glucose 6-phosphate dehydrogenase, GGT: gamma glutamyl transpeptidase, GK: glucokinase, GLUT: glucose transporter type, GPx: glutathione peroxidase, GSH: reduced glutathione, HDL: high-density lipoprotein, IFNγ: Interferon gamma, IL: interleukin, iNOS: inducible nitric oxide synthase, IR A: Insulin receptor A, LDH: lactate dehydrogenase, LDL: low-density lipoprotein, MMP: matrix metalloproteinase, NF-κB: nuclear factor κ-light-chain-enhancer of activated B cells, NO: nitric oxide, NTPDase: ectonucleoside triphosphate diphosphohydrolase-1, PKLR: pyruvate kinase, PLA: poly-lactide acid, PLGA: poly-(lactide-co-glycolide), PTP1B: protein tyrosine phosphatase 1B, PVA: polyvinyl alcohol, ROS: reactive oxygen species, SOD: superoxide dismutase, TCM: traditional Chinese medicine, TNF-α: tumour necrosis factor alpha, VLDL: very low-density lipoprotein.

References

1. Jini, D.; Sharmila, S. Green synthesis of silver nanoparticles from Allium cepa and its in vitro antidiabetic activity. Mater. Today Proc. 2019, https://doi.org/10.1016/j.matpr.2019.07.672.
2. Das, G.; Patra, J.K.; Debnath, T.; Ansari, A.; Shin, H.S. Investigation of antioxidant, antibacterial, antidiabetic, and cytotoxicity potential of silver nanoparticles synthesized using the outer peel extract of *Ananas comosus* (L.). *PloS one*. **2019**, *14*, e0220950.
3. Kumar, N. Biocompatible silver nanoparticles synthesized from *Andrographis echioides* and its antioxidant and antidiabetic effects on STZ induced diabetic rats. *Int. J. Chemtech. Res*. **2018**, *11*, 265-283.
4. Rajakumar, G.; Thiruvengadam, M.; Mydhili, G.; Gomathi, T.; Chung, I.M. Green approach for synthesis of zinc oxide nanoparticles from *Andrographis paniculata* leaf extract and evaluation of their antioxidant, anti-diabetic, and anti-inflammatory activities. *Bioprocess Biosyst. Eng*. **2018**, *41*, 21-30.

5. Arbianti, R.; Dewi, V.; Imansari, F.; Hermansyah, H.; Sahlan, M. Nano-preparation of *Andrographis paniculata* extract by casein micelle for antidiabetic agent. *AIP Conf. Proc.* **2017**, *1817*, 030010.
6. Sudirman, S.; Hsu, Y.H.; Johnson, A.; Tsou, D.; Kong, Z.L. Amelioration effects of nanoencapsulated triterpenoids from petri dish-cultured *Antrodia cinnamomea* on reproductive function of diabetic male rats. *Int. J. Nanomedicine* **2018,** *13*, 5059-5073.
7. Saratale, G.D.; Saratale, R.G.; Benelli, G.; Kumar, G.; Pugazhendi, A.; Kim, D.S.; Shin, H.S. Anti-diabetic potential of silver nanoparticles synthesized with *Argyreia nervosa* leaf extract high synergistic antibacterial activity with standard antibiotics against foodborne bacteria. *J. Clust. Sci*. **2017**, *28*, 1709-1727.
8. Das, S.K.; Behera, S.; Patra, J.K.; Thatoi, H. Green synthesis of sliver nanoparticles using *Avicennia officinalis* and *Xylocarpus granatum* extracts and in vitro evaluation of antioxidant, antidiabetic and anti-inflammatory activities. *J. Clust. Sci.* **2019**, *30*, 1103-1113.
9. Rehana, D.; Mahendiran, D.; Kumar, R.S.; Rahiman, A.K. In vitro antioxidant and antidiabetic activities of zinc oxide nanoparticles synthesized using different plant extracts. *Bioprocess Biosyst. Eng*. **2017**, *40*, 943-957.
10. Bhagwat, T.R.; Joshi, K.A.; Parihar, V.S.; Asok, A.; Bellare, J.; Ghosh, S. Biogenic copper nanoparticles from medicinal plants as novel antidiabetic nanomedicine. *World J. Pharm. Res*. **2018**, *7*, 183-196.
11. Johnson, P.; Krishnan, V.; Loganathan, C.; Govindhan, K.; Raji, V.; Sakayanathan, P.; Vijayan, S.; Sathishkumar, P.; Palvannan, T. Rapid biosynthesis of *Bauhinia variegata* flower extract-mediated silver nanoparticles: an effective antioxidant scavenger and α-amylase inhibitor. *Artif. Cells Nanomed. Biotechnol*. **2018**, *46*, 1488-1494.
12. Janakiraman, V.; Jenifer, M.; Ramarajan, S. Anti diabetic and anti cancer effects of silver nanoparticles synthesised from *Botryodiplodia theobromae*-an endophytic fungi isolated from *Euphorbia hirta*-a weed. *Int. J. Pharm. Sci. Rev. Res*. **2018**, *53*, 72-76.
13. Nithiya, S.; Sangeetha, R. Amylase inhibitory potential of silver nanoparticles biosynthesized using *Breynia retusa* leaf extract. *World J. Pharm. Res*. **2014**, *3*, 1055-1066.
14. Anandhi, D.; Kanaga, G.; Jagatheesh, S.; Revathi, K. In vitro evaluation of biological activity (anti-inflammatory, antiarthritic, and antidiabetic) of green synthesized silver nanoparticles from ethanolic extract of *Chromolaena odorata*, *Caesalpinia coriaria* (bark and leaves). *Drug Invent. Today*. **2019**, *12*, 1267-1271.
15. Govindappa, M.; Hemashekhar, B.; Arthikala, M.K.; Rai Ravishankar, V.; Ramachandra, Y.L. Characterization, antibacterial, antioxidant, antidiabetic, anti-inflammatory and antityrosinase activity of green synthesized silver nanoparticles using *Calophyllum tomentosum* leaves extract. *Results Phys*. **2018**, *9*, 400-408.
16. Daisy, P.; Saipriya, K. Biochemical analysis of *Cassia fistula* aqueous extract and phytochemically synthesized gold nanoparticles as hypoglycemic treatment for diabetes mellitus. *Int. J. Nanomedicine*. **2012**, *7*, 1189-1202.
17. Prada, A.L.; Keita, H.; de Souza, T.P.; Lima, E.S.; Acho, L.D.R.; da Silva, M.J.A.; Carvalho, J.C.T.; Amado, J.R.R. *Cassia grandis* Lf nanodispersion is a hypoglycemic product with a potent α-glucosidase and pancreatic lipase inhibitor effect. *Saudi Pharm. J*. **2019**, *27*, 191-199.
18. Liu, Y.; Zeng, S.; Liu, Y.; Wu, W.; Shen, Y.; Zhang, L.; Li, C.; Chen, H.; Liu, A.; Shen, L.; Hu, B.; Wang, C. Synthesis and antidiabetic activity of selenium nanoparticles in the presence of polysaccharides from *Catathelasma ventricosum*. *Int. J. Biol. Macromol*. **2018**, *114*, 632-639.
19. Barkat, M.A.; Mujeeb, M. Comparative study of *Catharanthus roseus* extract and extract loaded chitosan nanoparticles in alloxan induced diabetic rats. *Int. I. Pharm. Biomed*. **2013**, *4*, 670-678.
20. Wilson, S.; Cholan, S.; Vishnu, U.; Sannan, M.; Jananiya, R.; Vinodhini, S.; Manimegalai, S.; Devi Rajeswari, V. In vitro assessment of the efficacy of free-standing silver nanoparticles isolated from *Centella asiatica* against oxidative stress and its antidiabetic activity. *Der. Pharmacia Lettre*. **2015**, *7*, 194-205.
21. Ponnanikajamideen, M.; Rajeshkumar, S.; Vanaja, M.; Annadurai, G. In vivo type 2 diabetes and wound-healing effects of antioxidant gold nanoparticles synthesized using the insulin plant *Chamaecostus cuspidatus* in albino rats. *Can. J. Diabetes*. **2019**, *43*, 82-89.
22. Elobeid, M.A. Amelioration of streptozotocin induced diabetes in rats by eco-friendly composite nano-cinnamon extract. *Pakistan J. Zool*. **2016**, *48*, 645-650.

23. Kouame, K.; Peter, A.I.; Akang, E.N.; Moodley, R.; Naidu, E.C.; Azu, O.O. Histological and biochemical effects of *Cinnamomum cassia* nanoparticles in kidneys of diabetic Sprague-Dawley rats. *Bosn. J. Basic. Med. Sci*. **2019**, *19*, 138-145.
24. Maddinedi, S.B.; Mandal, B.K.; Maddili, S.K. Biofabrication of size controllable silver nanoparticles - A green approach. *J. Photochem. Photobiol. B*. **2017**, *167*, 236-241.
25. Prasad, A.R.; Basheer, S.M.; Williams, L.; Joseph, A. Highly selective inhibition of α-glucosidase by green synthesised ZnO nanoparticles - In-vitro screening and in-silico docking studies. *Int. J. Biol. Macromol*. **2019**, *139*, 712-718.
26. Popli, D.; Anil, V.; Subramanyam, A.B.; Namratha, M.N.; Ranjitha, V.R.; Rao, S.N.; Rai, R.V.; Govindappa, M. Endophyte fungi, *Cladosporium* species-mediated synthesis of silver nanoparticles possessing in vitro antioxidant, anti-diabetic and anti-Alzheimer activity. *Artif. Cells Nanomed. Biotechnol*. **2018**, *46*, 676-683.
27. Yakoob, A.T.; Tajuddin, N.B.; Hussain, M.I.M.; Mathew, S.; Govindaraju, A.; Qadri, I. Antioxidant and hypoglycemic activities of *Clausena anisata* (Willd.) Hook F. Ex Benth. root mediated synthesized silver nanoparticles. *Pharmacogn. J*. **2016**, *8*, 579-586.
28. Arsia Tarnam, Y.; Nargis Begum, T.; Muhammad Ilyas, M.H.; Mathew, S.; Govindaraju, A.; Qadri, I. Green synthesis, antioxidant potential and hypoglycemic effect of silver nanoparticles using ethanolic leaf extract of *Clausena anisata* (Willd.) Hook. F. Ex Benth. of Rutaceae. *Pharmacogn. J*. **2016**, *8*, 565-575.
29. Vishnu Kiran, M.; Murugesan, S. Biological synthesis of silver nanoparticles from marine alga *Colpomenia sinuosa* and its in vitro anti-diabetic activity. *The American Journal of Bio-pharmacology Biochemistry and Life Sciences* **2014**, *3*, 1-7.
30. Tahir, H.; Rahman, J.; Ashraf, M.; Anjum, S.; Hussain, I.; Warsi, M.F. Seasonal variation's effect on antidiabetic activity of silver nanoparticles. *Bioinspir. Biomim. Nan*. **2019**, https://doi.org/10.1680/jbibn.18.00018.
31. Vinotha, V.; Iswarya, A.; Thaya, R.; Govindarajan, M.; Alharbi, N.S.; Kadaikunnan, S.; Khaled, J.M.; Al-Anbr, M.N.; Vaseeharan, B. Synthesis of ZnO nanoparticles using insulin-rich leaf extract: Anti-diabetic, antibiofilm and anti-oxidant properties. *J. Photochem. Photobiol. B*. **2019**, *197*, 111541.
32. Velumani, S. Green synthesis of gold nanoparticles from *Costus igneus*. *Int. J. Adv. Res. Innov. Ideas. Educ*. **2015**, *1*, 972-978.
33. Aruna, A.; Nandhini, R.; Karthikeyan, V.; Bose, P.; Vijayalakshmi, K. Comparative anti-diabetic effect of methanolic extract of insulin plant (*Costus pictus*) leaves and its silver nanoparticle. *Indo. Am. J. Pharm. Sci*. **2014**, *4*, 3217-3230.
34. Alamoudi, E.F.; Khalil, W.K.B.; Ghaly, I.S.; Hassan, N.H.A.; Ahmed, E.S. Nanoparticles from of *Costus speciosus* extract improves the antidiabetic and antilipidemic effects against STZ-induced diabetes mellitus in albino rats. *Int. J. Pharm. Sci. Rev. Res*. **2014**, *29*, 279-288.
35. Surya, C.; John, N.A.A.; Pandiyan, V.; Ravikumar, S.; Amutha, P.; Sobral, A.J.F.N.; Krishnakumar, B. *Costus speciosus* leaf extract assisted CS-Pt-TiO2 composites: Synthesis, characterization and their bio and photocatalytic applications. *J. Mol. Struct*. **2019**, *1195*, 787-795.
36. Sengani, M.; Devi Rajeswari, V. Identification of potential antioxidant indices by biogenic gold nanoparticles in hyperglycemic Wistar rats. *Environ. Toxicol. Pharmacol*. **2017**, *50*, 11-19.
37. Kumar, D.G.; Ninganna, D.; Kantharaju, R.M. Antioxidant, antibacterial, antidiabetic potential and genotoxicity of silver nanoparticles using leaf extract of *Curcuma longa*: a novel green approach. *Int. Res. J. Pharm*. **2019**, *10*, 127-136.
38. Agarwal, H.; Kumar, S.V.; Rajeshkumar, S. Antidiabetic effect of silver nanoparticles synthesized using lemongrass (*Cymbopogon citratus*) through conventional heating and microwave irradiation approach. *J. Microbiol. Biotechnol. Food Sci*. **2018**, *7*, 371-376.
39. Ghosh, S.; More, P.; Nitnavare, R.; Jagtap, S.; Chippalkatti, R.; Derle, A.; Kitture, R.; Asok, A.; Kale, S.; Singh, S.; Shaikh, M.L.; Ramanamurthy, B.; Bellare, J.; Chopade, B.A. Antidiabetic and Antioxidant Properties of Copper Nanoparticles Synthesized by Medicinal Plant *Dioscorea bulbifera*. *J. Nanomed. Nanotechnol.* **2015**, *S6*, 007.
40. Al Rashid, H. Preparation and characterization of plga loaded nanoparticles obtained from *D. melanoxylon* Roxb. leaves for their antiproliferative and antidiabetic activity. *International Journal of Green Pharmacy* **2017**, *11*, S438-S447.

41. Kunjiappan, S.; Panneerselvam, T.; Prasad, P.; Sukumaran, S.; Somasundaram, B.; Sankaranarayanan, M.; Murugan, I.; Parasuraman, P. Design, graph theoretical analysis and in silico modeling of *Dunaliella bardawil* biomass encapsulated keratin nanoparticles: a scaffold for effective glucose utilization. *Biomed. Mater*. **2018**, *13*, 045012.
42. Senthikumar, P.; Kumar, D.S.R.S.; Sudhagar, B.; Vanthana, M.; Parveen, M.H.; Sarathkumar, S.; Thomas, J.C.; Mary, A.S.; Kannan, C. Seagrass-mediated silver nanoparticle synthesis by *Enhalus acoroides* and its α-glucosidase inhibitory activity from the Gulf of Mannar. *J. Nanostructure Chem.* **2016**, *6*, 275-280.
43. Garcia Campoy, A.H.; Perez Gutierrez, R.M.; Manriquez-Alvirde, G.; Muñiz Ramirez, A. Protection of silver nanoparticles using *Eysenhardtia polystachya* in peroxide-induced pancreatic β-cell damage and their antidiabetic properties in zebrafish. *Int. J. Nanomedicine* **2018**, *13*, 2601-2612.
44. Jamdade, D.A.; Rajpali, D.; Joshi, K.A.; Kitture, R.; Kulkarni, A.S.; Shinde, V.S.; Bellare, J.; Babiya, K.R.; Ghosh, S. *Gnidia glauca*- and *Plumbago zeylanica*-mediated synthesis of novel copper nanoparticles as promising antidiabetic agents. *Adv. Pharmacol. Sci*. **2019**, *2019*, 9080279.
45. Venkatachalam, M.; Govindaraju, K.; Mohamed Sadiq, A.; Tamilselvan, S.; Ganesh Kumar, V.; Singaravelu, G. Functionalization of gold nanoparticles as antidiabetic nanomaterial. *Spectrochim Acta A Mol. Biomol. Spectrosc*. **2013**, *116*, 331-338.
46. Karthick, V.; Kumar, V.G.; Dhas, T.S.; Singaravelu, G.; Sadiq, A.M.; Govindaraju, K. Effect of biologically synthesized gold nanoparticles on alloxan-induced diabetic rats-an in vivo approach. *Colloids Surf B Biointerfaces* **2014**, *122*, 505-511.
47. Shanker, K.; Krishna Mohan, G. Anti-diabetic and hypo-lipidemic activity of green synthesized silver nanoparticles and *G. sylvestre* extract on streptozotocin induced diabetic rats: An in vivo approach. *Endocrino.l Metab. Syndr*. **2018**, *7*, 44.
48. Nirmala, S.; Ravichandiran, V.; Vijayalakshmi, A.; Nadanasabapathi, P. Protective effect of *Gymnemic acid* isolated from *Gymnema sylvestre* leaves coated chitosan reduced gold nanoparticles in hyperlipedimia and diabetes induced vascular tissue damage in rats. *Res. J. Pharm. Tech*. **2018**, *11*, 1193-1206.
49. Vishnu Kiran, M.; Murugesan, S. Biogenic silver nanoparticles by *Halymenia poryphyroides* and its in vitro anti-diabetic efficacy. *J. Chem. Pharm. Res*. **2013**, *5*, 1001-1008.
50. Aravinthan, A.; Kamala-Kannan, S.; Govarthanan, M.; Kim, J.H. Accumulation of biosynthesized gold nanoparticles and its impact on various organs of Sprague Dawley rats: a systematic study. *Toxicol Res (Camb)*. **2016**, *5*, 1530-1538.
51. Thatoi, P.; Kerry, R.G.; Gouda, S.; Das, G.; Pramanik, K.; Thatoi, H.; Patra, J.K. Photo-mediated green synthesis of silver and zinc oxide nanoparticles using aqueous extracts of two mangrove plant species, *Heritiera fomes* and *Sonneratia apetala* and investigation of their biomedical applications. *J. Photochem. Photobiol. B*. **2016**, *163*, 311-318.
52. Bala, N.; Saha, S.; Chakraborty, M.; Maiti, M.; Das, S.; Basub, R.; Nandyc, P. Green synthesis of zinc oxide nanoparticles using *Hibiscus subdariffa* leaf extract: effect of temperature on synthesis, anti-bacterial activity and anti-diabetic activity. *RSC Adv*. **2015**, *5*, 4993-5003.
53. Kumar, V.; Singh, S.; Srivastava, B.; Bhadouria, R.; Singh, R. Green synthesis of silver nanoparticles using leaf extract of *Holoptelea integrifolia* and preliminary investigation of its antioxidant, anti-inflammatory, antidiabetic and antibacterial activities. *J. Environ. Chem. Eng*. **2019**, *7*, 103094.
54. Das, G.; Patra, J.K.; Basavegowda, N.; Vishnuprasad, C.N.; Shin, H.S. Comparative study on antidiabetic, cytotoxicity, antioxidant and antibacterial properties of biosynthesized silver nanoparticles using outer peels of two varieties of *Ipomoea batatas* (L.) Lam. *Int. J. Nanomedicine* **2019**, *14*, 4741-4754.
55. Kalakotla, S.; Jayarambabu, N.; Mohan, G.K.; Mydin, R.B.S.M.N.; Gupta, V.R. A novel pharmacological approach of herbal mediated cerium oxide and silver nanoparticles with improved biomedical activity in comparison with *Lawsonia inermis*. *Colloids Surf. B Biointerfaces*. **2019**, *174*, 199-206.
56. Debnath, G.; Das, P.; Saha, A.K. Characterization, antimicrobial and α-amylase inhibitory activity of silver nanoparticles synthesized by using mushroom extract of *Lentinus tuber-regium*. *Proc Natl Acad Sci India, Sect B Biol Sci*. **2019**, https://doi.org/10.1007/s40011-019-01076-y.
57. Balan, K.; Qing, W.; Wang, Y.; Liu, X.; Palvannan, T.; Wang, Y.; Ma, F.; Zhang, Y. Antidiabetic activity of silver nanoparticles from green synthesis using *Lonicera japo*nica leaf extract. *RSC Adv*. **2016**, *6*, 40162-40168.

58. Mathur, R.; Vijayvergia, R. Green synthesis of silver nanoparticles by using leaf and stem of *Manilkara zapota* L. and antioxidant and antidiabetic activity. *Int. J. Biol. Med. Res*. **2017**, *8*, 6142-6145.
59. Chowdhury, A.; Kunjiappan, S.; Bhattacharjee, C.; Somasundaram, B.; Panneerselvam, T. Biogenic synthesis of *Marsilea quadrifolia* gold nanoparticles: a study of improved glucose utilization efficiency on 3T3-L1 adipocytes. *In Vitro Cell Dev. Biol. Anim*. **2017**, *53*, 483-493.
60. Shanker, K.; Naradala, J.; Mohan, G.K.; Kumar, G.S.; Pravallika, P.L. A sub-acute oral toxicity analysis and comparative in vivo anti-diabetic activity of zinc oxide, cerium oxide, silver nanoparticles, and *Momordica charantia* in streptozotocin-induced diabetic Wistar rats. *RSC Adv*. **2017**, *7*, 37158-37167.
61. Krishna Mohan, G.; Shanker, K. Biologically synthesized silver nanoparticles from *Momordica charantia* and assessment of antidiabetic activity on Streptozotocin induced diabetic rats. *Medicinal Plants*. **2017**, *6*, 57.
62. Soliman, N.A.; Ismail, E.H.; El-Moaty, H.I.A.; Sabry, D.Y.; Khalil, M.M.H. Anti-*Helicobacter pylori*, anti-diabetic and cytotoxicity activity of biosynthesized gold nanoparticles using *Moricandia Nitens* water extract. *Egypt J. Chem*. **2018**, 61(4), 691-703.
63. Rajan, A.R.; Rajan, A.; John, A.; Philip, D. Green synthesis of CeO_2 nanostructures by using *Morus nigra* fruit extract and its antidiabetic activity. *AIP Conference Proceedings*. **2019**, *2105*, 020008.
64. Deng, W.; Wang, H.; Wu, B.; Zhang, X. Selenium-layered nanoparticles serving for oral delivery of phytomedicines with hypoglycemic activity to synergistically potentiate the antidiabetic effect. *Acta Pharm. Sin. B*. **2019**, *9*, 74-86.
65. Bayrami, A.; Alioghli, S.; Pouran, S.R.; Habibi-Yangjeh, A.; Khataee, A.; Ramesh, S. A facile ultrasonic-aided biosynthesis of ZnO nanoparticles using *Vaccinium arctostaphylos* L. leaf extract and its antidiabetic, antibacterial, and oxidative activity evaluation. *Ultrasonics Sonochemistry* **2019**, *55*, 57-66.
66. Triastuti, E.; Yurina, V.; Yunita, E.P. Effect of *Nigella Sativa* encapsulated in poly (lactide-co-glycolide) nanoparticles on plasma malondialdehyde and ischemia-modified albumin in a diabetes rat model. *Journal of Pharmaceutical Research & Clinical Practice*. **2016**, *6*, 1-7.
67. Kavitha, K.; Sujatha, K.; Manoharan, S. Development, characterization and antidiabetic potentials of *Nilgirianthus ciliatus* Nees derived nanoparticles. *J. Nanomedine Biotherapeutic Discov*. **2017**, *7*, 152.
68. Malapermal, V.; Botha, I.; Krishna, S.B.N.; Mbatha, J.N. Enhancing antidiabetic and antimicrobial performance of *Ocimum basilicum*, and *Ocimum sanctum* (L.) using silver nanoparticles. *Saudi J. Biol. Sci*. **2017**, *24*, 1294-1305.
69. Malapermal, V.; Mbatha, J.N.; Gengan, R.M.; Anand, K. Biosynthesis of bimetallic Au-Ag nanoparticles using *Ocimum basilicum* (L.) with antidiabetic and antimicrobial properties. *Adv. Mater. Lett*. **2015**, *6*, 1050-1057.
70. Senthilkumar, P.; Priya, L.; Kumar, R.S.; Bhuvaneshwari, D.S. Potent α-glucosidase inhibitory activity of green synthesized gold nanoparticles from the brown seaweed *Padina boergesenii*. *International Journal of Recent Advances in Multidisciplinary Research* **2015**, *2*, 917-923.
71. Leela, K.; Anchana Devi, C. study on the applications of silver nanoparticle synthesized using the aqueous extract and the purified secondary metabolites of lichen *Parmelia perlata*. *Int. J. Pharm. Sci. Invent*. **2017**, *6*, 42-59.
72. Al-Twaty, N.H.; Booles, H.F. Nano-encapsulated form of *Phyllanthus emblica* extract increases its therapeutic effects as antidiabetic and antioxidant in rats. *Int. J. Pharm. Sci. Rev. Res*. **2014**, *29*, 11-17.
73. Patra, J.K.; Das, G.; Shin, H.S. Facile green biosynthesis of silver nanoparticles using *Pisum sativum* L. outer peel aqueous extract and its antidiabetic, cytotoxicity, antioxidant, and antibacterial activity. *Int. J. Nanomedicine* **2019**, *14*, 6679-6690.
74. Debnath, G.; Das, P.; Saha, A.K. Green synthesis of silver nanoparticles using mushroom extract of P*leurotus giganteus*: characterization, antimicrobial, and α-amylase inhibitory activity. *BioNanoScience* **2019**, *9*, 611-619.

75. Aldawsari, H.M.; Hanafy, A.; Labib, G.S.; Badr, J.M. Antihyperglycemic activities of extracts of the mistletoes *Plicosepalus acaciae* and *P. curviflorus* in comparison to their solid lipid nanoparticle suspension formulations. *Z. Naturforsch C*. **2014**, *69*, 391-398.
76. Prabhu, S.; Vinodhini, S.; Elanchezhiyan, C.; Rajeswari, D. Evaluation of antidiabetic activity of biologically synthesized silver nanoparticles using *Pouteria sapota* in streptozotocin-induced diabetic rats. *J. Diabetes*. **2018**, *10*, 28-42.
77. Shanker, K.; Mohan, G.K.; Hussain, M.A.; Jayarambabu, N.; Pravallika, P.L. Green biosynthesis, characterization, in vitro antidiabetic activity, and investigational acute toxicity studies of some herbal-mediated silver nanoparticles on animal models. *Pharmacogn. Mag*. **2017**, *13*, 188-192.
78. Bagyalakshmi, J.; Haritha, H. Green synthesis and characterization of silver nanoparticles using *Pterocarpus marsupium* and assessment of its in vitro antidiabetic activity. *Am. J. Adv. Drug. Deliv*. **2017**, *5*, 118-130.
79. Kitture, R.; Chordiya, K.; Gaware, S.; Ghosh, S.; More, P.A.;, Kulkarni, P.; Chopade, B.A.; Kale, S.N. ZnO nanoparticles-red sandalwood conjugate: a promising anti-diabetic agent. *J. Nanosci. Nanotechnol*. **2015**, *15*, 4046-4051.
80. Saratale, R.G.; Shin, H.S.; Kumar, G.; Benelli, G.; Kim, D.S.; Saratale, G.D. Exploiting antidiabetic activity of silver nanoparticles synthesized using *Punica granatum* leaves and anticancer potential against human liver cancer cells (HepG2). *Artif. Cells. Nanomed. Biotechnol*. **2018**, *46*, 211-222.
81. Virgen-Ortiz, A.; Limón-Miranda, S.; Soto-Covarrubias, M.A.; Apolinar-Iribe, A.; Rodríguez-León, E.; Iñiguez-Palomares, R. Biocompatible silver nanoparticles synthesized using *Rumex hymenosepalus* extract decreases fasting glucose levels in diabetic rats. *Dig. J. Nanomater. Bios*. **2015**, *10*, 927-933.
82. Opris, R.; Tatomir, C.; Olteanu, D.; Moldovan, R.; Moldovan, B.; David, L.; Nagy, A.; Decea, N.; Kiss, M.L. Filip, G.A. The effect of *Sambucus nigra* L. extract and phytosinthesized gold nanoparticles on diabetic rats. *Colloids Surf B Biointerfaces* **2017**, *150*, 192-200.
83. Patra, N.; Kar, D.; Pal, A.; Behera, A. Antibacterial, anticancer, anti-diabetic and catalytic activity of bio-conjugated metal nanoparticles. *Adv. Nat. Sci.: Nanosci. Nanotechnol*. **2018**, *9*, 035001.
84. Dhas, T.S.; Kumar, V.G.; Karthick, V.; Vasanth, K.; Singaravelu, G.; Govindaraju, K. Effect of biosynthesized gold nanoparticles by *Sargassum swartzii* in alloxan induced diabetic rats. *Enzyme Microb. Technol*. **2016**, *95*, 100-106.
85. Bano, F.; Baber, M.; Ali, A.; Shah, Z.; Muhammad, S.A. Biosynthesis, characterization, and biological activities of iron nanoparticles using *Sesamum indicum* seeds extract. *Pharmacogn. Mag*. **2017**, *13*, S33-S36.
86. Mohammadi Arvanag, F.; Bayrami, A.; Habibi-Yangjeh, A.; Rahim Pouran, S. A comprehensive study on antidiabetic and antibacterial activities of ZnO nanoparticles biosynthesized using *Silybum marianum* L seed extract. *Mater. Sci. Eng. C. Mater. Biol. Appl*. **2019**, *97*, 397-405.
87. Sengottaiyan, A.; Aravinthan, A.; Sudhakar, C.; Selvam, K.; Srinivasan, P.; Govarthanan, M.; Manoharan, K.; Selvankumar, T. Synthesis and characterization of *Solanum nigrum*-mediated silver nanoparticles and its protective effect on alloxan-induced diabetic rats. *J. Nanostructure Chem*. **2016**, *6*, 41-48.
88. Swarnalatha, L.; Rachel, C.; Ranjan, S.; Baradwaj, P. Evaluation of in vitro antidiabetic activity of *Sphaeranthus amaranthoides* silver nanoparticles. International *Journal of Nanomaterials and Biostructures* **2012**, *2*, 25-29.
89. Perumal, V.; Manickam, T.; Bang, K.S.; Velmurugan, P.; Oh, B.T. Antidiabetic potential of bioactive molecules coated chitosan nanoparticles in experimental rats. *Int. J. Biol. Macromol*. **2016**, *92*, 63-69.
90. Langle, A.; González-Coronel, M.A.; Carmona-Gutiérrez, G.; Moreno-Rodríguez, J.A.; Venegas, B.; Munoz, G.; Trevino, S.; Díaz, A. *Stevia rebaudiana* loaded titanium oxide nanomaterials as an antidiabetic agent in rats. *Rev. Bras. Farmacogn*. **2015**, *25*, 145-151.
91. Sabatini, P.; Anchana Devi, C. Anti-microbial, ovicidal, larvicidal, anti-diabetic and cytoxicity activity of silver nanoparticles from brown seaweed (*Stoechospermum marginatum*). *Int. J. Pharm. Sci. Res*. **2018**, *9*, 1555-1564.

92. Atale, N.; Saxena, S.; Nirmala, J.G.; Narendhirakannan, R.T.; Mohanty, S.; Rani, V. Synthesis and characterization of *Sygyzium cumini* nanoparticles for its protective potential in high glucose-induced cardiac stress: a green approach. *Appl. Biochem. Biotechnol*. **2017**, *181*, 1140-1154.
93. Bitencourt, P.E.R.; dos Santos Stein, C.; Borges, R.M.; Moretto, M.B. Aqueous seed extract of *Syzygium cumini* (asc) and polymeric nanoparticles (npasc) modulate adenine nucleotide and nucleoside hydrolysis in diabetic rats with candidiasis. *Diabetol. Metab. Syndr*. **2015**, *7*, A2.
94. Samadder, A.; Das, S.; Das, J.; Paul, A.; Khuda-Bukhsh, A.R. Ameliorative effects of *Syzygium jambolanum* extract and its poly (lactic-co-glycolic) acid nano-encapsulated form on arsenic-induced hyperglycemic stress: a multi-parametric evaluation. *Journal of acupuncture and meridian studies* **2012**, *5*, 310-318.
95. Hima Bindu, R.; Mohana Lakshmi, S.; Himaja, N.; Nirosha, K.; Pooja, M. Formulation, characterisation and antidiabetic evaluation of *Talinum portulacifolium* (Forssk.) loaded solid lipid nanoparticles in streptozotocin & high fat diet induced diabetic rats. *Journal of Global Trends in Pharmaceutical Sciences* **2014,** *5*, 2108-2114.
96. Rajaram, K.; Aiswarya, D.C.; Sureshkumar, K. Green synthesis of silver nanoparticle using *Tephrosia tinctoria* and its antidiabetic activity. *Mater. Lett*. **2015**, *138*, 251-254.
97. Ragavee, A.; Devi, S.A. Nanoencapsulation of *Tinospora cordifolia* (Willd.) using poly (D, L-lactide) nanoparticles: yield optimization by response surface methodology and in silico modeling with insulin receptor tyrosine kinase. *Phcog. Mag*. **2019**, *15*, 218-227.
98. Walvekar, M.V.; Pol, S.B.; Deshmukh, V.M. Modulatory effect of fenugreek loaded PLGA nanoparticles on lipofuscinogenesis in pancreas of alloxan induced diabetic mice. *International Journal of Pharmaceutical and Clinical Research* **2016**, *8*, 22-25.
99. Virk, P. Antidiabetic activity of green gold-silver nanocomposite with *Trigonella foenum graecum* L. seeds extract on streptozotocin induced diabetic rats. *Pakistan J. Zool*. **2018**, *50*, 711-718.
100. Bayrami, A.; Parvinroo, S.; Habibi-Yangjeh, A.; Rahim Pouran, S. Bio-extract-mediated ZnO nanoparticles: microwave-assisted synthesis, characterization and antidiabetic activity evaluation. *Artif. Cells Nanomed. Biotechnol*. **2018**, *46*, 730-739.
101. Sampathkumar, K.; Riyajan, S.; Tan, C.K.; Demokritou, P.; Chudapongse, N.; Loo, S.C.J. Small-intestine-specific delivery of antidiabetic extracts from *withania coagulans* using polysaccharide-based enteric-coated nanoparticles. *ACS Omega* **2019**, *4*, 12049-12057.
102. Li, Y.; Zhang, J.; Gu, J.; Chen, S.; Wang, C.; Jia, W. Biosynthesis of polyphenol-stabilised nanoparticles and assessment of anti-diabetic activity. *J. Photochem. Photobiol. B*. **2017**, *169*, 96-100.
103. Uddin, M.I.; Kavya, G.; Soundarya, G.; Ropa, G.; Sridhar, C.H. Anti-diabetic activity of silver nanoparticles synthesized from ashwagandha root aqueous extract. *Asian Journal of Pharmaceutical Education and Research* **2018**, *7*, 45-59.
104. Sun, Z.; Lu, F.; Cheng, J.; Zhang, M.; Zhu, Y.; Zhang, Y.; Kong, H.; Qu, H.; Zhao, Y. Hypoglycemic bioactivity of novel eco-friendly carbon dots derived from traditional Chinese medicine. *J. Biomed. Nanotechnol*. **2018**, *14*, 2146-2155.
105. Lin, D.; Du, Q.; Wang, H.; Gao, G.; Zhou, J.; Ke, L.; Chen, T.; Shaw, C.; Rao, P. Antidiabetic micro-/nanoaggregates from Ge-Gen-Qin-Lian-Tang decoction increase absorption of baicalin and cellular antioxidant activity in vitro. *Biomed. Res. Int*. **2017**, *2017*, 9217912.
106. Umrani, R.D.; Paknikar, K.M. Jasada bhasma, a zinc-based Ayurvedic preparation: contemporary evidence of antidiabetic activity inspires development of a nanomedicine. *Evid. Based Complement. Alternat. Med*. **2015**, *2015*, 193156.
107. Hossein-Nia, B.; Khorram, S.; Rezazadeh, H.; Safaiyan, A.; Ghiasi, R.; Tarighat-Esfanjani, A. The effects of natural clinoptilolite and nano-sized clinoptilolite supplementation on lipid profile, food intakes and body weight in rats with streptozotocin-induced diabetes. *Adv. Pharm. Bull*. **2018**, *8*, 211-216.
108. Hossein-Nia, B.; Khorram, S.; Rezazadeh, H.; Safaiyan, A.; Tarighat-Esfanjani, A. The effects of natural clinoptilolite and nano-sized clinoptilolite supplementation on glucose levels and oxidative stress in rats with type 1 diabetes. *Can. J. Diabetes*. **2018**, *42*, 31-35.
